# PKM2 deficiency exacerbates gram-negative sepsis-induced cardiomyopathy via disrupting cardiac calcium homeostasis

**DOI:** 10.1038/s41420-022-01287-9

**Published:** 2022-12-23

**Authors:** Le Ni, Bowen Lin, Meiting Shen, Can Li, Lingjie Hu, Fengmei Fu, Lei Chen, Jian Yang, Dan Shi

**Affiliations:** 1 Department of Cardiology, Shanghai East Hospital, Tongji University School of Medicine, Shanghai, 200120 China; 2 Key Laboratory of Arrhythmias of the Ministry of Education of China, Shanghai East Hospital, Tongji University School of Medicine, Shanghai, 200120 China; 3 Jinzhou Medical University, Liaoning, 121000 China; 4 Shanghai Frontiers Science Center of Nanocatalytic Medicine, Shanghai, 200092 China

**Keywords:** Cardiomyopathies, Cardiovascular diseases

## Abstract

Sepsis is a life-threatening syndrome with multi-organ dysfunction in critical care medicine. With the occurrence of sepsis-induced cardiomyopathy (SIC), characterized by reduced ventricular contractility, the mortality of sepsis is boosted to 70–90%. Pyruvate kinase M2 (PKM2) functions in a variety of biological processes and diseases other than glycolysis, and has been documented as a cardioprotective factor in several heart diseases. It is currently unknown whether PKM2 influences the development of SIC. Here, we found that PKM2 was upregulated in cardiomyocytes treated with LPS both in vitro and in vivo. *Pkm2* inhibition exacerbated the LPS-induced cardiac damage to neonatal rat cardiomyocytes (NRCMs). Furthermore, cardiomyocytes lacking PKM2 aggravated LPS-induced cardiomyopathy, including myocardial damage and impaired contractility, whereas PKM2 overexpression and activation mitigated SIC. Mechanism investigation revealed that PKM2 interacted with sarcoplasmic/endoplasmic reticulum calcium ATPase 2a (SERCA2a), a key regulator of the excitation-contraction coupling, to maintain calcium homeostasis, and PKM2 deficiency exacerbated LPS-induced cardiac systolic dysfunction by impairing SERCA2a expression. In conclusion, these findings highlight that PKM2 plays an essential role in gram-negative sepsis-induced cardiomyopathy, which provides an attractive target for the prevention and treatment of septic cardiomyopathy.

## Introduction

Gram-negative bacteria are the leading cause of sepsis [1], a systemic inflammatory response syndrome that is estimated to cause millions of death worldwide per year [2]. Lipopolysaccharide (LPS) is the main component of the outer membrane of gram-negative bacteria, which is the key factor in stimulating host resistance to infection and is the central pathogenesis of gram-negative sepsis [3]. Sepsis-induced cardiomyopathy (SIC) is one of the most devastating manifestations, which is also an important predictor of poor prognosis [4]. The mortality of patients suffering SIC is raised to 70–90% [5, 6]. Aside from uncontrolled inflammatory responses, the factors associated with the development of SIC include dysregulated calcium homeostasis, excessive apoptosis, abnormal mitochondria, oxidative stress and aberrant autophagy, etc. [7–9]. However, effective therapies for SIC are still lacking due to an incomplete understanding of its pathogenesis.

It has been reported that the mortality of septic patients is directly associated with poor cardiac contractility [10–12]. Cardiac contraction depends on the release and uptake of calcium by sarcoplasmic reticulum of cardiomyocytes, indicating that calcium homeostasis is essential for the maintenance of normal myocardium contraction-relaxation cycle [13–15]. Moreover, recent studies revealed that disrupted calcium homeostasis in SIC caused cardiomyocyte apoptosis, which was accompanied by oxidative stress and inflammatory responses, ultimately impairing systolic and diastolic functions of the heart [16, 17]. Thus, improving cardiac systolic function may be an effective therapeutic strategy in SIC.

Pyruvate kinase M2 (PKM2), an isoform of the PKM gene, participates in multiple cellular processes such as migration, proliferation, apoptosis, and immune responses [18–20]. PKM1 and PKM2 are highly diverse in protein structure and function, although there is only one single exon difference in their coding sequences [21, 22]. To date, PKM2 has been associated with various human diseases, such as autoimmune diseases [23], cancer [24], dysplasia [25] and viral infection [26]. More recent evidence from our group and others suggested that PKM2 deficiency severely compromised cardiac function and prognosis in cardiac disorders, and that regulating PKM2 expression may be an effective way to alleviate these pathological processes [25, 27, 28]. However, the relationship between PKM2 and calcium homeostasis in SIC is largely unknown. Given the import role of PKM2 in cardiovascular function, this study aimed to understand the impact of PKM2 on cardiac function in SIC.

Herein, our data showed that PKM2 expression was activated in cardiomyocytes in response to LPS stimulation, whereas PKM2 deletion exacerbated LPS-induced cardiomyopathy. In contrast, PKM2 overexpression and activation significantly ameliorated SIC. Mechanistically, PKM2 deficiency impaired cardiac contraction by modulating sarcoplasmic/endoplasmic reticulum calcium ATPase 2a (SERCA2a) against LPS attack. Altogether, we revealed that PKM2 protects cardiac contraction in LPS-induced SIC by regulating SERCA2a, which may serve as a potential therapeutic target for SIC.

## Results

### PKM2 expression increased in cardiomyocytes in response to lipopolysaccharide (LPS)

**Fig. 1:**
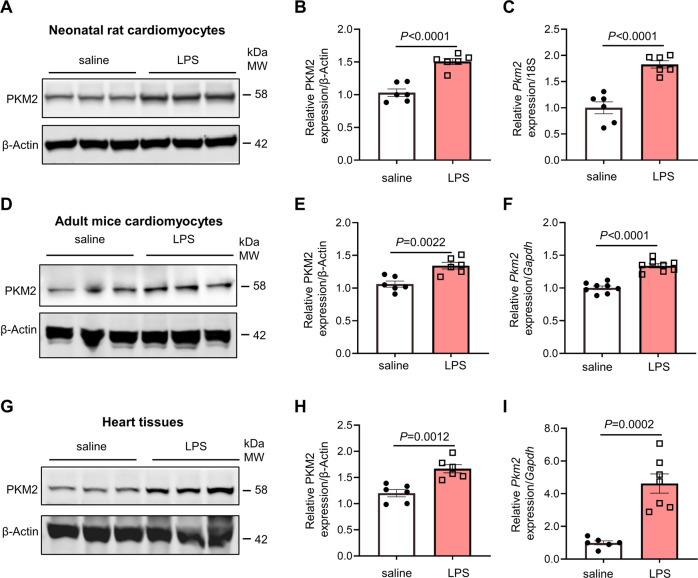
PKM2 expression increased in cardiomyocytes in response to lipopolysaccharide (LPS). **A**, **B** Western blot and quantification of PKM2 expression in neonatal rat cardiomyocytes (NRCMs) with saline or LPS administration for 24 h. β-Actin served as loading control, *n* = 6. **C** Quantitative real-time polymerase chain reaction (qRT-PCR) analysis of *Pkm2* in NRCMs administrated with LPS. *18S* was used as an internal reference gene, *n* = 6. **D**, **E** Western blot and quantification of cardiac PKM2 expression in AMCMs administrated with saline or LPS for 24 h. β-Actin served as loading control, *n* = 6. **F** qRT-PCR analysis of *Pkm2* in AMCMs administrated with LPS. *Gapdh* was used as an internal reference gene, *n* = 8. **G**, **H** Western blot and quantification of cardiac PKM2 expression in myocardial tissues. β-Actin served as loading control, *n* = 6. **I** qRT-PCR analysis of *Pkm2* in myocardial tissues. *Gapdh* was used as an internal reference gene, *n* = 6 to 7. Values represent the mean ± SEM of at least three independent experiments.

To examine PKM2 expression in cardiomyocytes in LPS-induced cardiomyopathy, we firstly purified neonatal rat cardiomyocytes (NRCMs) and adult mouse cardiomyocytes (AMCMs) following 24 h LPS (10 μg/mL) administration. PKM2 mRNA and protein expression were significantly increased after LPS administration than those treated with saline (Fig. 1A–F). Considering PKM1 and PKM2 are alternative splicing products derived from the *Pkm* gene, we also assessed the expression of PKM1 in NRCMs and AMCMs. Unlike PKM2, PKM1 expression remained unchanged in response to LPS (Fig. S1A–C). Next, we investigated PKM2 expression in hearts from the mice exposed to LPS. Mice were randomly injected either LPS (10 mg/kg) or saline intraperitoneally. Compared with saline control, both the protein and mRNA expression of PKM2 was higher in LPS-challenged animals (Fig. 1G–I). As indicated in Fig. S1, there was no difference in the expression of PKM1 between the control and LPS-challenged animals, consistent with the in vitro findings on cardiomyocytes. These results indicated that PKM2 may participate in the development of LPS-induced cardiomyopathy.

### PKM2 deficiency in cardiomyocytes exacerbated LPS-induced cardiomyopathy in vitro and in vivo

**Fig. 2:**
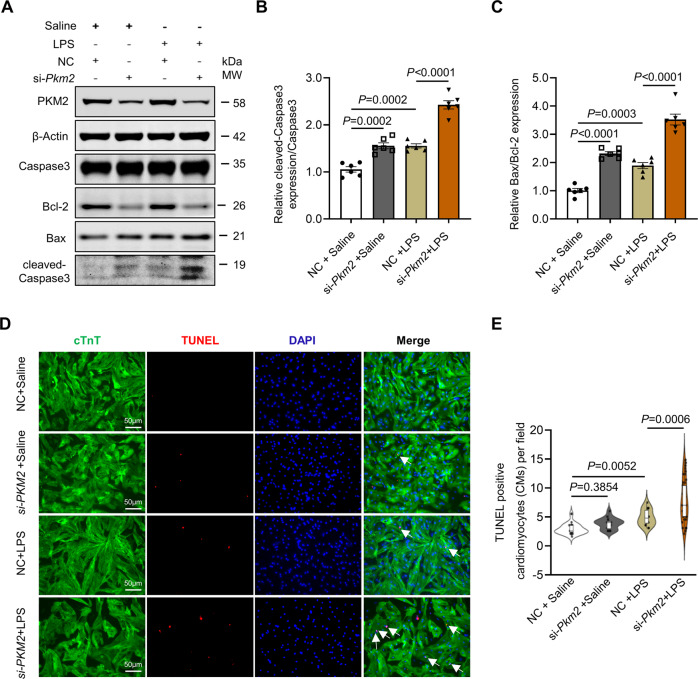
PKM2 deficiency in cardiomyocytes exacerbates LPS-induced cardiomyopathy in vitro. **A**–**C** Western blot and quantitation of apoptosis markers (cleaved-Caspase 3/Caspase 3 and Bax/Bcl-2 ratio) expression in NRCMs co-administered with *Pkm2* siRNA and LPS. β-Actin served as loading control, *n* = 6. **D**, **E** Representative images and quantification of TUNEL staining from four groups (NC+Saline, *n* = 28; si-*Pkm2*+Saline, *n* = 26; NC+LPS, *n* = 25; si-*Pkm2*+LPS, *n* = 50; scale bar, 50 μm). White arrows point to TUNEL^+^ cells. NC negative control. Values represent the mean ± SEM of at least three independent experiments.

Given that LPS induces apoptosis of cardiomyocytes independent of stimulators released by non-cardiomyocytes [29], we employed apoptosis as an indicator to evaluate LPS-induced myocardial injury. First, to explore the role of PKM2 in the development of LPS-induced cardiomyopathy in vitro, we knocked down *Pkm2* in NRCMs by small interfering RNAs (siRNA). Remarkably, *Pkm2* deficiency resulted in apparently elevated expression of apoptosis markers (cleaved-Caspase 3/Caspase3 and Bax/Bcl2 ratio), which was further exacerbated after LPS treatment (Fig. 2A–C). Then, we performed another apoptosis assay-TUNEL staining to validate the increased expression of apoptosis markers in *Pkm2* knockdown NRCMs after LPS treatment. As shown in Fig. 2D, E, the number of TUNEL positive cardiomyocytes was obviously increased post LPS administration, which was consistent with previous studies [30, 31]. Besides, there was only a slight increase in TUNEL positive cardiomyocytes in *Pkm2* deficient cardiomyocytes, while significantly more TUNEL positive cardiomyocytes were detected in *Pkm2* deficient cardiomyocytes upon LPS stimulation.

**Fig. 3:**
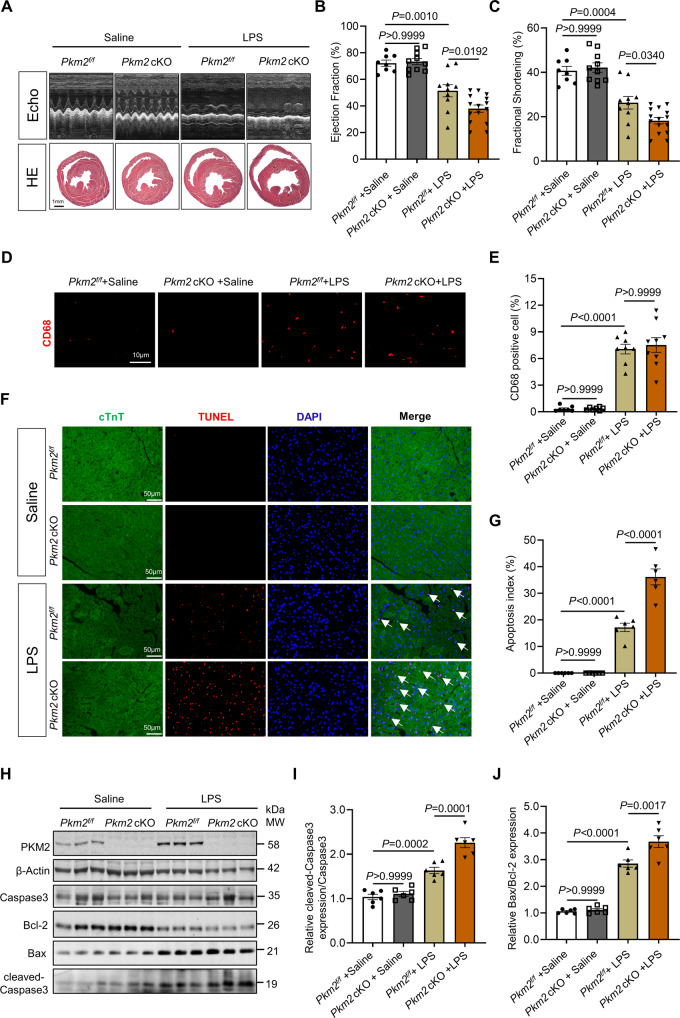
PKM2 deficiency in cardiomyocytes exacerbates LPS-induced cardiomyopathy in vivo. **A** Representative M-mode echocardiography and heart cross-sections stained with hematoxylin-eosin (HE) (scale bar, 1 mm) of *Pkm2* cKO and *Pkm2*^*f/f*^ mice 24 h after saline or LPS administration. **B**, **C** Quantitative analysis of ejection fraction (EF) and fractional shortening (FS) by echocardiography, *n* = 8 to 15. **D**, **E** Representative images of CD68 and statistical analysis, *n* = 7 to 9. Scale bar: 10 μm. **F**, **G** Representative images and quantification of TUNEL staining, *n* = 6. Scale bar: 50 µm. White arrows point to TUNEL positive cardiomyocytes. **H**, **I** Western blot and quantitation of apoptosis markers (cleaved-Caspase 3/Caspase 3 and Bax/Bcl-2 ratio) expression in hearts of *Pkm2* cKO and *Pkm2*^*f/f*^ mice 24 h after saline or LPS administration. β-Actin served as loading control, *n* = 6. Values represent the mean ± SEM of at least three independent experiments.

Next, we set to evaluate the role of PKM2 in vivo. We generated cardiomyocyte-specific *Pkm2* conditional knockout mice (*Pkm2* cKO) by crossing α-MHC-Cre mice with *Pkm2*^*f/f*^ mice. *Pkm2*^*f/f*^ mice were used as controls. As we previously reported that there were no differences in heart development and basal cardiac functions between the *Pkm2* cKO mice and their controls [28]. The *Pkm2*^*f/f*^ and *Pkm2* cKO mice were divided randomly into four groups (*Pkm2*^*f/f*^-saline, *Pkm2* cKO-saline, *Pkm2*^*f/f*^-LPS, and *Pkm2* cKO-LPS). Our data showed that injection of LPS for 24 h contributed to the systolic dysfunction (evaluated based on ejection fraction (EF), fractional shortening (FS), and other parameters), and *Pkm2* knockout in cardiomyocytes further exacerbated this adverse effect (Figs. 3A–C and S2A–D). Hematoxylin-eosin (HE) staining further displayed dilated heart chambers in *Pkm2* cKO-LPS versus *Pkm2*^*f/f*^-LPS mice (Fig. 3A). Given the role of PKM2 in regulating inflammation, we sought to investigate the effects of cardiomyocytic PKM2 on Gram-negative bacteria associated cardiomyopathy. First, renowned pro-inflammatory cytokines such as IL-6, TNF-α and IL-1β were analyzed by quantitative real-time polymerase chain reaction (qRT-PCR) under LPS treatment. As shown in Fig. S3A–C, LPS promoted the levels of the pro-inflammatory cytokines (IL-6, TNF-α and IL-1β), but there was no difference between *Pkm2*^*f/f*^ and *Pkm2* cKO mice in response to LPS. Consistently, immunohistochemistry analysis revealed enhanced CD68, a macrophage marker, expression in mice challenged with LPS, while there was no significant difference between *Pkm2*^*f/f*^ and *Pkm2* cKO mice (Fig. 3D, E). The results above indicated that PKM2 deficiency in cardiomyocytes rarely affected heart inflammatory after LPS stimulation. We then analyzed cardiac apoptosis, the typical feature of LPS-induced cardiomyopathy. First, in both saline injected *Pkm2*^*f/f*^ and *Pkm2* cKO mice, TUNEL staining showed little cardiac apoptosis. However, after LPS administration, aggravated apoptosis was observed in hearts, which was significantly more severe in *Pkm2* cKO mice (Fig. 3F, G). Concomitantly, expression of cleaved-Caspase 3/Caspase3 and Bax/Bcl-2 ratio showed a similar trend as TUNEL staining in heart tissues (Fig. 3H–J). Taken together, these data demonstrated that PKM2-deficient cardiomyocytes exacerbate cardiac function and myocardial injury after LPS treatment, suggesting an increased susceptibility to LPS-induced cardiomyopathy in mice.

### PKM2 interacted with SERCA2a and regulated its expression

**Fig. 4:**
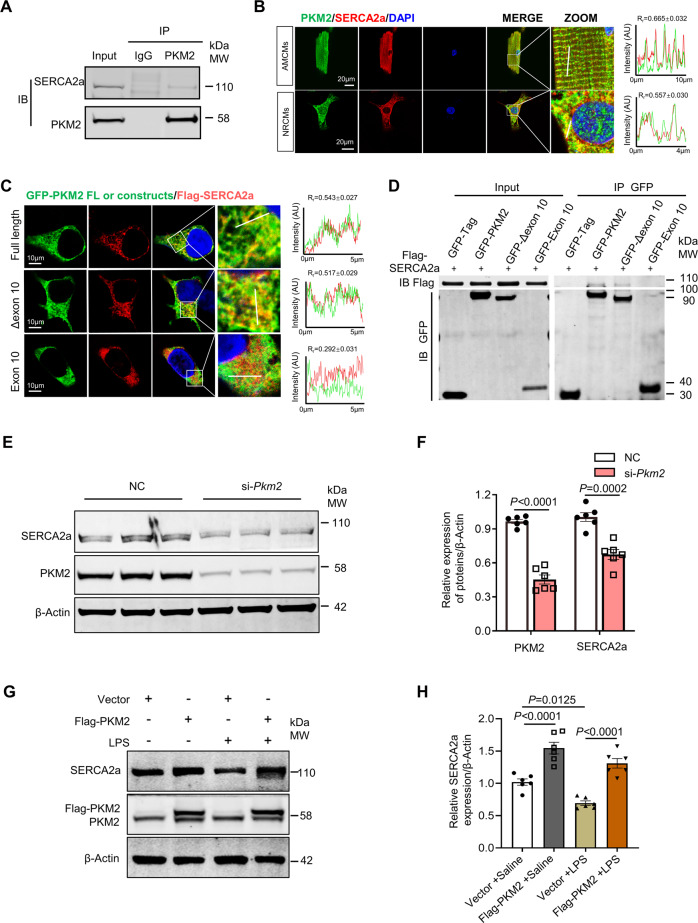
PKM2 interacted with SERCA2a and regulated its expression. **A** Immunoprecipitation assay to validate SERCA2a using anti-PKM2 antibody in NRCMs. **B** Representative immunofluorescence images showing the co-localization of PKM2 (green) and SERCA2a (red) in AMCMs and NRCMs by confocal immunofluorescence analysis (scale bars, 20 μm). Line profile analyses showing the distribution and intensity of fluorescence signals. The Pearson’s coefficient was measured from the images using ImageJ software (*n* = 18). **C** Immunofluorescence analysis of the colocalization of exogenous-expressing SERCA2a (red) and PKM2 full-length and truncated forms. The right column indicates the line plot profiles of the region of interest (*n* = 15). **D** Co-immunoprecipitation by anti-GFP antibody (PKM2 full-length and truncated forms) with Flag tagged SERCA2a in transiently transfected HEK-293T cells. **E**, **F** Western blot and quantitation of SERCA2a and PKM2 expression in NRCMs after *Pkm2* knockdown. β-Actin served as loading control, *n* = 6. **G**, **H** Western blot and quantitation of SERCA2a expression in NRCMs co-administered with PKM2 overexpressing plasmid and LPS. β-Actin served as loading control, *n* = 6. Values represent the mean ± SEM of at least three independent experiments.

Since LPS impairs cardiac contractility and disrupts calcium homeostasis directly [32, 33], we assessed related proteins involved in cardiac calcium handling and contractility (PLB, RyR2, CaMKII and SERCA2a). There was no significant difference in expression of these proteins between *Pkm2*^*f/f*^ and *Pkm2* cKO mice treated with saline. While after LPS administration, only SERCA2a expression was significantly decreased in *Pkm2*^*f/f*^ mice and reduced even further in *Pkm2* cKO mice (Fig. S4A–E). However, no difference at *Serca2a* mRNA level was detected between *Pkm2*^*f/f*^ and *Pkm2* cKO mice after LPS treatment (Fig. S4F). These data indicated that PKM2 post-transcriptionally affected the SERCA2a expression during LPS-induced cardiomyopathy. Then, we set to elucidate the relationship between PKM2 and SERCA2a. We performed PKM2 immunoprecipitation (IP) in NRCMs, and Western blot validated SERCA2a as a potential partner of PKM2 (Fig. 4A). We then examined their cellular localization by immunofluorescence to determine the relationship between PKM2 and SERCA2a in situ. Consistent with IP result, confocal microscopy analysis revealed that the cytoplasmic co-localization of PKM2 and SERCA2a in both NRCMs and AMCMs (Fig. 4B).

PKM2 differs from PKM1 only by the alternative splicing of exon 10 (Fig. S5A) [22]. To understand the structural basis for the interaction between PKM2 and SERCA2a in detail, we constructed plasmids containing *Pkm2* full-length, exon 10 only and a truncated mutant (exon 10 deletion, *Pkm2*-Δexon 10) for further investigation (Fig. S5B). GFP tagged *Pkm2* full-length, exon 10 or *Pkm2*-Δexon 10 were co-transfected with Flag-SERCA2a into HEK-293T cells, and visualized by immunofluorescence. Among the two truncated forms tested, only the *Pkm2*-Δexon 10 mutant was able to co-localize with SERCA2a (Fig. 4C), Consistent with immunofluorescence results, IP and Western blot revealed that PKM2 interacted with SERCA2a outside exon 10 (Fig. 4D). We then examined the interaction between PKM1 and SERCA2a. Intriguingly, PKM1 did not interact with SERCA2a by immunoprecipitation and confocal microscopy assays (Fig. S5C, D), suggesting that the exon 9 prevent PKM1 from interacting with SERCA2a. Remarkably, *Pkm2* knockdown caused a significant decrease in SERCA2a protein expression in NRCMs (Fig. 4E, F). Considering that LPS is able to inhibit SERCA2a expression in cardiomyocytes [34, 35], we examined whether PKM2 overexpression could alleviate LPS-induced SERCA2a reduction in cardiomyocytes. Our data showed that SERCA2a was downregulated in LPS treated NRCMs and partially restored to the level close to saline control after PKM2 overexpression (Fig. 4G, H).

### PKM2 deficiency worsened cardiomyocyte contraction amplitude, calcium transients upon LPS stimulation

**Fig. 5:**
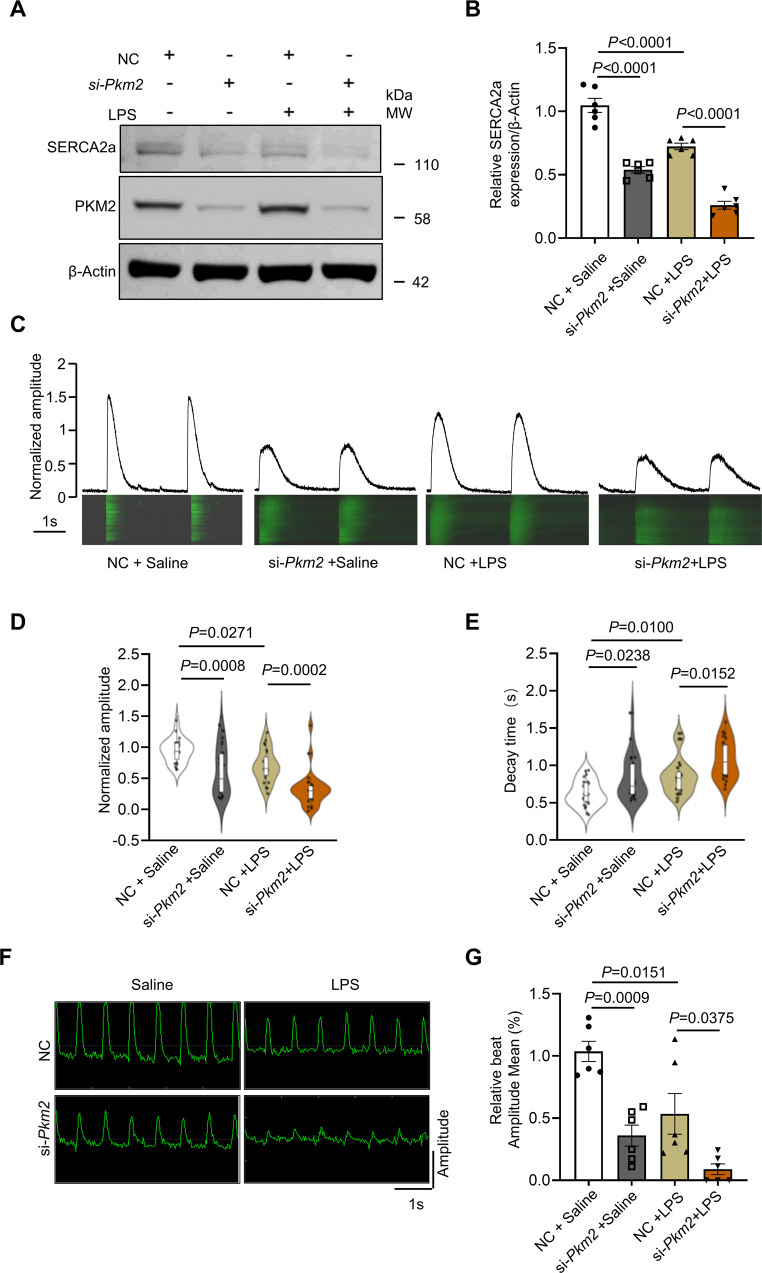
PKM2 knockdown compromised cardiomyocyte contraction amplitude, calcium transients in NRCMs. **A**, **B** Western blot and quantitation of SERCA2a and PKM2 expression in NRCMs co-administered with *Pkm2* siRNA and LPS. β-Actin served as loading control, *n* = 6. **C**–**E** Representative calcium transient curve and quantitation of calcium transient amplitudes and decay time in NRCMs, *n* = 24. **F**, **G** Assessment of the contractility of NRCMs co-administered with *Pkm2* siRNA and LPS. Contractility waveform shows contraction amplitude of cardiomyocytes, *n* = 6. NC negative control. Values represent the mean ± SEM of at least three independent experiments.

SERCA2a is a critical pump responsible for intracellular calcium handling, which regulates cardiomyocyte function, especially contractility [36]. We, therefore, went to investigate the role of PKM2 in dynamic calcium transient and cardiomyocyte contractility. As mentioned above, *Pkm2* deficient cardiomyocytes displayed low level of SERCA2a protein, which was further reduced upon LPS stimulation (Fig. 5A, B). Concomitantly, Pkm2 knockdown resulted in a slower calcium transient decay rate and a reduction in calcium transient amplitude when compared to scrambled siRNA-transfected cardiomyocytes. Moreover, LPS treatment significantly exacerbated *Pkm2* deficiency-induced prolongation of the decay phase duration and reduction in the calcium transient amplitude (Fig. 5C–E). To further assess the relationship between PKM2 and cardiac contractility, we then evaluated the contractility of NRCMs with the Maestro Pro and Maestro Edge microelectrode array (MEA) systems by employing the mean beat amplitude to describe the contractile behavior of the cardiomyocytes. After *Pkm2* knockdown, the cardiac contractility of NRCMs, as reflected by mean beat amplitude, decreased significantly, which was further exacerbated after LPS treatment (Fig. 5F, G). Collectively, these data suggested that PKM2 deficiency hampered cardiomyocyte contraction amplitude and calcium transients in vitro, which was consistent with SERCA2a downregulation.

**Fig. 6:**
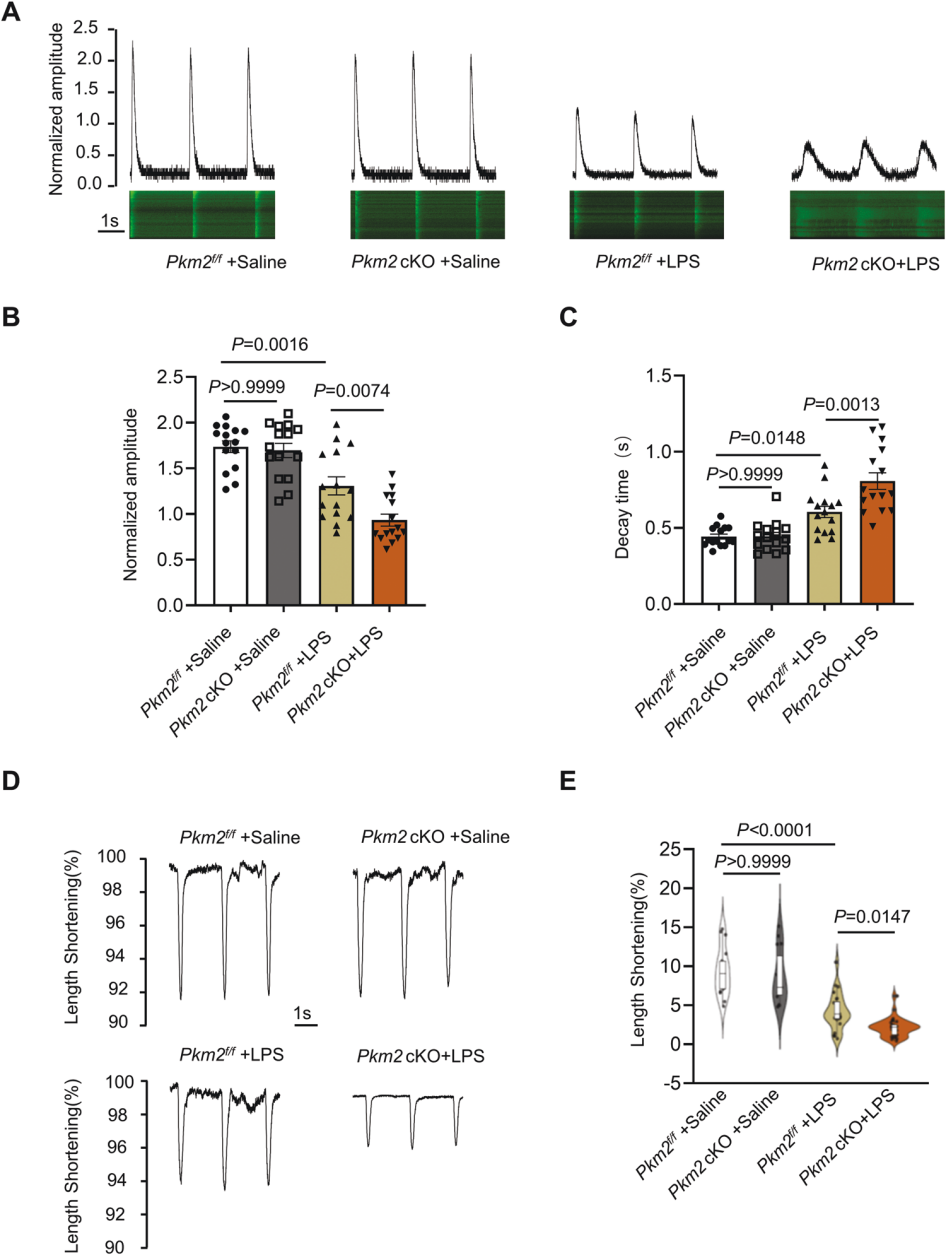
PKM2 deficiency compromised cardiomyocyte contraction amplitude, calcium transients. **A**–**C** Representative calcium transient curve and quantitation of calcium transient amplitudes and decay time in AMCMs from *Pkm2* cKO and *Pkm2*^*f/f*^ mice after saline or LPS administration, *n* = 15. **D**, **E** Cardiomyocytes contraction depicting cell shortening in cardiomyocytes isolated from *Pkm2* cKO and *Pkm2*^*f/f*^ mice after saline or LPS administration, *n* = 24. Values represent the mean ± SEM.

Next, we validated these findings in LPS-induced mouse cardiomyopathy model. AMCMs were purified from mice in four groups (*Pkm2*^*f/f*^-saline, *Pkm2* cKO-saline, *Pkm2*^*f/f*^-LPS, and *Pkm2* cKO-LPS) to explore the alteration in calcium handling. Surprisingly, there was no significant difference in calcium transient decay rate and amplitude between *Pkm2*^*f/f*^ and *Pkm2* cKO mice injected with saline. After intraperitoneal injection of LPS, the calcium transient amplitude in AMCMs from *Pkm2* cKO mice was significantly reduced compared with that from *Pkm2*^*f/f*^ mice, and the calcium transient decay rate was significantly extended in *Pkm2* cKO than that in *Pkm2*^*f/f*^ mice (Fig. 6A–C). Subsequently, we further examined systolic function, which was determined by sarcomere shortening, and found that PKM2 knockout aggravated the decline of sarcomere shortening in *Pkm2*^*f/f*^ mice exposed to LPS (Fig. 6D, E). Taken together, we concluded that PKM2 deficiency leads to SERCA2a downregulation, which in turn disrupted calcium homeostasis and impaired myocardial contractility.

### PKM2 gain of function study in cardiomyocytes improved cardiac function after cecum ligation and puncture (CLP)-induced cardiomyopathy

**Fig. 7:**
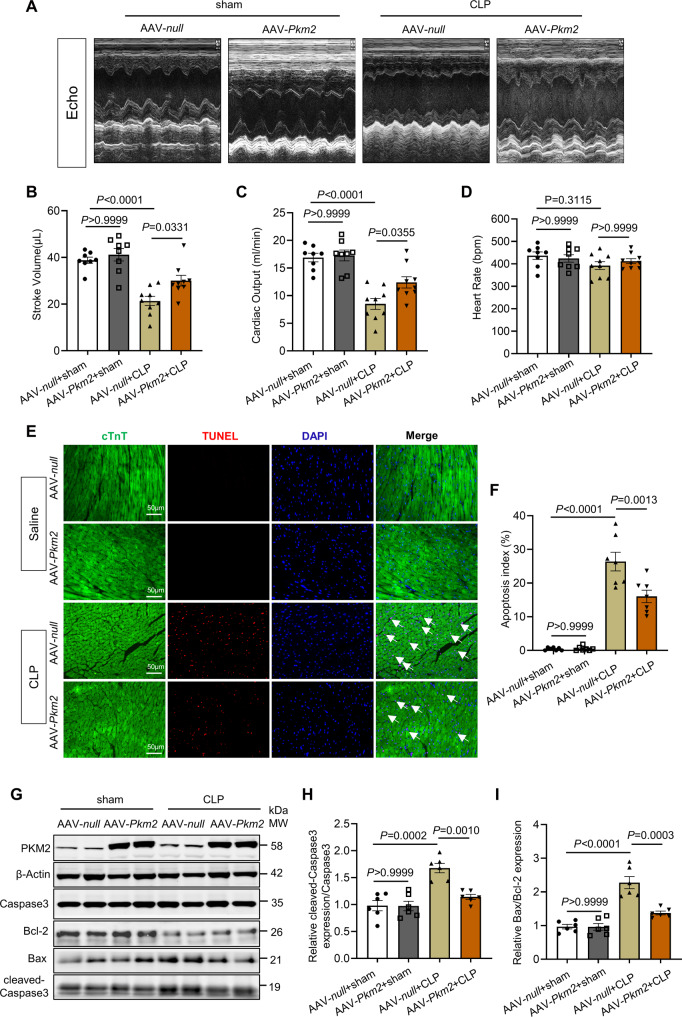
PKM2 overexpression in cardiomyocytes alleviated cecum ligation and puncture (CLP)-induced cardiomyopathy in vivo. **A** Representative M-mode echocardiography from littermate injected AAV9-*null* or AAV9-*Pkm2* virus after CLP surgery. **B**–**D** Quantitative analysis of stroke volume (SV), cardiac output (CO), and heart rate by echocardiography, *n* = 8. **E**, **F** Representative images and quantification of TUNEL staining, *n* = 7. Scale bar: 50 µm. White arrows point to TUNEL positive cardiomyocytes. **G**–**I** Western blot and quantitation of apoptosis markers (cleaved-Caspase 3/Caspase3 and Bax/Bcl-2 ratio) expression in hearts of littermate injected AAV9-*null* or AAV9-*Pkm2* virus after CLP surgery. β-Actin served as the loading control, *n* = 6.

Recent studies have reported that overexpression and agonist of PKM2 can mitigate several heart diseases, such as DOX-induced cardiomyopathy, myocardial infarction, etc. [25, 27]. To determine whether PKM2 exhibits protective effects in SIC, we employed a clinically relevant sepsis mouse model, cecum ligation and puncture (CLP), and adeno-associated virus (AAV)-cardiac troponin T (cTnT) system to specifically overexpress of *Pkm2* in cardiomyocytes. The animals were divided into four groups (AAV-*null* + sham, AAV*-Pkm2* + sham, AAV*-null* + CLP and AAV*-Pkm2* + CLP). As shown in Fig. 7A–C, echocardiography revealed no differences in basal cardiac functions between the AAV-*null* + sham and AAV*-Pkm2* + sham mice. The stroke volume (SV) was significantly preserved in AAV*-Pkm2* + CLP than in AAV-*null* + CLP mice, and the cardiac output (CO) displayed similar results. Besides, the CLP had no significant effect on the mouse heart rate, suggesting that heart rate was not the cause of the difference in CO and SV between AAV*-null* + CLP and AAV*-Pkm2* + CLP mice (Fig. 7D). Then, we examined the apoptosis of cardiomyocytes 24 h after the CLP procedure. TUNEL staining showed little cardiac apoptosis in both AAV*-null* + sham and AAV*-Pkm2* + sham mice. However, aggravated apoptosis was observed in CLP-operated hearts, which was much alleviated in AAV*-Pkm2* + CLP mice (Fig. 7E, F). Consistent with the above TUNEL results, apoptosis markers (cleaved-Caspase 3/Caspase3 and Bax/Bcl2 ratio) were partially decreased in the *Pkm2* overexpression group, close to the level in the sham group after CLP operation (Fig. 7G–I).

**Fig. 8:**
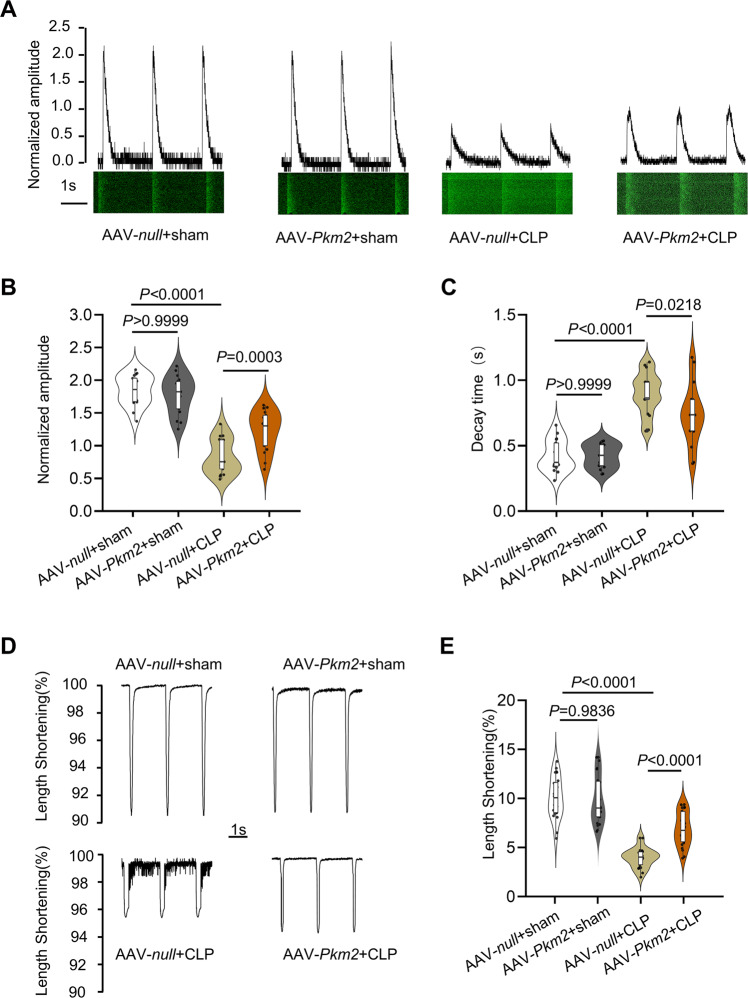
Cardiac-specific PKM2 overexpression protected cardiomyocytes from CLP-induced systolic disfunction. **A**–**C** Representative calcium transient curve and quantitation of calcium transient amplitudes and decay time in AMCMs from littermates injected AAV9-*null* or AAV9-*Pkm2* virus after CLP surgery, *n* = 17. **D**, **E** Cardiomyocytes contraction depicting cell shortening in cardiomyocytes isolated from littermate injected AAV9-*null* or AAV9-*Pkm2* virus after CLP surgery, *n* = 22–25. Values represent the mean ± SEM.

Considering the regulation of SERCA2a by PKM2, AMCMs were purified from four groups to explore the alteration in calcium handling and cardiomyocyte contractility. Though there was no significant difference in calcium transient decay rate and amplitude between AAV*-null* + sham and AAV-*Pkm2* + sham mice, the calcium transient amplitude in AMCMs from AAV*-null* + CLP mice was significantly reduced compared with that from AAV*-Pkm2* + sham mice, and the calcium transient decay rate was significantly extended in AAV*-null* + CLP than that in AAV*-Pkm2* + CLP mice (Fig. 8A–C). Concomitantly, PKM2 overexpression alleviated the declined systolic function of cardiomyocytes after CLP operation, which was determined by sarcomere shortening (Fig. 7D, E). Additionally, we utilized TEPP46, a small-molecule PKM2 agonist, to confirm the protective effect of PKM2 in SIC. Similar to PKM2 overexpression, TEPP46 diminished apoptosis and improved functional recovery in CLP-induced cardiomyopathy (Figs. S6 and S7). Collectively, these findings indicated that PKM2 gain of function contributed to the protective effect on CLP-induced mice cardiomyopathy.

## Discussion

In the present study, we found that PKM2 was upregulated in cardiomyocytes in response to LPS both in vitro and in vivo. Further investigations revealed that PKM2 deficiency aggravated LPS-induced cardiomyopathy, manifesting more severe myocardial injury and poorer cardiac contractility. Moreover, the development of sepsis-induced cardiomyopathy was ameliorated in PKM2 overexpression and activation mice. Mechanistically, for the first time, we uncovered that PKM2 interacted with SERCA2a to mediate calcium uptake into the sarcoplasmic reticulum, thereby maintaining the excitation–contraction coupling in cardiomyocytes. Collectively, these results provided a novel mechanistic insight into gram-negative sepsis-induced cardiomyopathy and a potential target for its intervention.

Inflammation has long been a research focus of SIC, including gram-negative infection induced [37]. However, numerous therapeutics targeting specific inflammatory cytokines were failed in clinical trials, emphasizing the urgent need to explore novel therapeutic target [38, 39]. The reason for this could be that LPS directly affects myocardial function and induces cardiomyocytes apoptosis, independent of inflammatory cytokines [40, 41]. Therefore, maintaining sufficient quantity of functional cardiomyocytes can thus prevent or significantly delay the deterioration of cardiac function during sepsis. The terminal outcomes of LPS-induced cardiomyopathy include calcium homeostasis imbalance, myocardial ultrastructural changes, and even cell death and contractile dysfunction [42, 43]. Although the expression of PKM2 is low in cardiomyocytes, multiple studies have demonstrated that PKM2 serves as a cardioprotective factor by interacting with proteins related to proliferation (e.g., β-catenin) [25] or apoptosis (e.g., P53) [27]. Here, we found that cardiomyocytic PKM2 was activated in response to LPS, and its deficiency aggravated LPS-induced cardiomyopathy. Our further investigation revealed that PKM2 regulates cardiac systole function by interacting with SERCA2a. These results emphasized that modulating PKM2 might be a potential new strategy to intervene LPS-induced cardiomyopathy.

Calcium homeostasis is essential for normal myocardium contraction/relaxation cycle in cardiomyocytes [15]. The systole of cardiomyocytes is triggered by the rhythm of calcium sparks released by RyR2, while the diastole is delicately regulated by SERCA2a. Since RyR2 and SERCA are two key factors responsible for calcium-handing between the cytosol and the SR/ER [15, 44], RyR2 dysfunction or SERCA2a downregulation/inactivation can impair cardiac pump function. Abnormal SERCA2a expression results in cytosolic calcium overload, depressed cardiomyocyte contractility and apoptosis [13]. It has been reported that decreased SERCA2a expression or activity was associated with poor prognosis and survival in LPS-induced cardiomyopathy [35, 45]. Conversely, the replenishments of SERCA2a had the opposite effect [35, 46]. However, the causes of SERCA2a dysregulation in LPS-induced cardiomyopathy have not been fully elucidated yet. In our research, cardiomyocytic PKM2 directly interacted with SERCA2a and regulated its protein expression. PKM2 deficiency resulted in the downregulation of SERCA2a, followed by calcium imbalance, cardiomyocyte apoptosis, and cardiac contractility impairment, which was further exacerbated after LPS treatment. Collectively, our data suggested that PKM2-SERCA2a-calcium axis may play an indispensable role in the regulation of myocardial contractility, especially in LPS-induced cardiomyopathy.

PKM is the key enzyme responsible for converting phosphoenolpyruvate to pyruvate at the final step of glycolysis [47]. PKM1 and PKM2 isoforms are alternative-splicing products of the PKM gene, including a mutually exclusive exon (exon 9 for PKM1 and exon 10 for PKM2) [22]. Although the interaction between PKM2 with SERCA2a was via exon 1–8 or 11–12, the same domains as PKM1, we confirmed that PKM1 did not interact with SERCA2a. The reason is that PKM1 and PKM2 differ greatly in their spatial structure, subcellular location, and biochemical functions, due to the difference of only 22 amino acids encoded by exon 10 [21, 48, 49]. PKM2 functions indispensably for the expression of SERCA2a and its exon 10 was the critical structure.

There are still several aspects that need further consideration. On the one hand, our study lacks results from human heart samples, and this may limit the applicability of the findings to the initiation and development of SIC in human. On the other hand, further research is needed to find ways to regulate PKM2, which may contribute to precision heart disease therapy.

In summary, our study unveils the role of PKM2 in gram-negative sepsis-induced cardiomyopathy. PKM2 deficiency leads to cardiomyocytes systolic dysfunction and causes subsequently poor prognosis in SIC. Moreover, PKM2 overexpression and activation protected cardiac function from SIC. These new findings will advance our understanding of the pathogenesis of gram-negative sepsis-induced cardiomyopathy and provide a novel perspective for the mechanism of intracellular SERCA2a regulation.

## Materials and methods

### Animals

All animal procedures were approved by the Ethics Committee of Tongji University School of Medicine. All mice were on the C57BL/6 background and were kept under specific-pathogen-free (SPF) conditions with a 12:12 h light-dark cycle at 24 ± 2 °C and 45–65% humidity. Cardiomyocyte-specific *Pkm2* conditional knockout (*Pkm2* cKO) mice were generated as described previously [50]. In brief, the mice with *Pkm2* gene exon 10 flanked by two loxP sites (*Pkm2*^*f/f*^) were purchased from the Jackson Laboratory (024048) and backcrossed to the C57BL/6 background for more than 6 generations before crossed with the αMHC-Cre transgenic mice. Genotyping primers are listed in Table S1.

### *Pkm2* overexpression experiments

An adeno-associated virus type 2/9 (AAV2/9)-cardiac troponin T (cTnT) system was chosen for the delivery of cardiomyocyte-specific genes. Recombinant AAV2/9-cTnT-EGFP and AAV2/9-cTnT-PKM2-P2A-EGFP viruses were synthesized in Taitool Co., Shanghai, China. Two weeks before CLP surgery, 50 μL/mouse (5.8 × 10^11^ viral particles) viruses were injected via the tail vein under anesthesia as previously described [51].

### Gram-negative sepsis-induced cardiomyopathy mouse model

In this study, gram-negative sepsis-induced cardiomyopathy model was established with lipopolysaccharide (LPS) (Sigma; L2280) or cecal ligation and puncture (CLP) [52, 53]. For LPS-induced cardiomyopathy, mice weighed 22.3 ± 2.5 g were randomly underwent an intraperitoneal injection of LPS (10 mg/kg) or saline equally. The general appearance and heart function of the animals were monitored. After LPS stimulation for 24 h, the mice were sacrificed for subsequent experiments. The CLP surgery procedure was similar to previous studies with minor modifications [52]. Briefly, mice were anesthetized with 2% isoflurane (RWD, R510-22-4) with a maintained 37 °C body temperature. After exposure through a small anterior abdominal incision, the cecum was ligated with a 4–0 silk suture and was punctured twice with an 18-gauge needle. The cecum was then gently squeezed to extrude a small amount of stool from the puncture site to ensure complete perforation. Next, the cecum was relocated into the peritoneal cavity, and the abdominal incision was closed with 6–0 silk sutures. Sham-operated mice underwent the same operation procedure but without CLP. Subsequent experiments were performed 24 h after the operation.

### Echocardiography

Echocardiography was performed by the Vevo 770 High-Resolution In Vivo Micro-Imaging System (VisualSonics, Vevo-770) before and 24 h after LPS stimulation. After mice were anesthetized by isoflurane (RWD, R510-22-4) inhalation through a mask, echocardiography parameters were measured under the long-axis M-mode with heart rate over 350 beats per minute, including left ventricular internal end-diastolic diameter (LVID, d), left ventricular internal end-systolic diameter (LVID, s), fractional shortening (FS), ejection fraction (EF) and other parameters.

### Plasmid construction

Plasmids encoding the homo-GFP-PKM2 and two truncated forms (exon 10 and *Pkm2*-Δexon 10: exon 10 deletion) were constructed as described previously [54]. Plasmid homo-GFP-PKM2 (Sino Biological, HG11430-ACG) was used as template to generate exon 10 and *Pkm2*-Δexon 10 by ClonExpress II One Step Cloning Kit (Vazyme, C112-01) and Phanta Max Super-Fidelity DNA Polymerase (Vazyme, P505-d1), based on homologous recombination method. The primers used are as follows: exon 10: 5′-CCACCAAGCTTGGTACCATGAGGTCTGCTCACCAGGTGGC-3′ (forward), 5′-CTCACAGAGCCTCCACCCCCGCCAACATTCATGGCAAAGTT-3′ (reverse). Primers for amplification of the vector were 5′-GGGGGTGGAGGCTCTGTG-3′ (forward), 5′-CATGGTACCAAGCTTGGTGGC-3′ (reverse). The primers for *Pkm2*-Δexon 10 were 5′-TCACCAAGTCTGGCAAGGCCCGAGGCTTCTTCA-3′ (forward), 5′-CCTTGCCAGACTTGGTGAGGACGATTATGGCCC-3′ (reverse).

### Cardiomyocyte isolation, culture, transfection and LPS treatment

The isolation and culture procedures for primary neonatal rat cardiomyocytes (NRCMs) and adult mouse cardiomyocytes (AMCMs) were modified from previous studies [55, 56]. The cardiomyocytes were transfected with small interference RNA (siRNA) against *Pkm2* (40 nmol/L) with lipofectamine™ RNAimax transfection reagent (Invitrogen, 13778150). The scrambled siRNA (40 nmol/L) was used as a negative control. Flag-PKM2 plasmid (Sino Biological, HG11430-CF) was transfected into NRCMs with lipofectamine® 3000 transfection reagent (Invitrogen, L3000015) according to the manufacturer’s protocols. The Flag-vector (Sino Biological, CV005) was used as a negative control. After knockdown or overexpression of PKM2 for 48 h, LPS (10 μg/mL) was added to the culture medium for another 24 h. Then cells were collected for subsequent analysis. The siRNA sequences were listed in Table S2.

### Calcium transients

Calcium transient images were captured by Lecia laser scanning confocal microscope (Lecia, SP5). Cardiomyocytes were first incubated in a 37 °C incubator with 5 µg/mL Calbryte™ 520 AM (AAT Bioquest, 20650) for 30–60 min, followed by incubation at room temperature (RT) for an additional 15 min. Cardiomyocytes were bathed in Hank’s balanced salt solution (HBSS) at RT, and systolic calcium transient was recorded under constant field stimulation at 0.5 Hz and 25 V with a confocal microscope.

### Measurement of cardiomyocyte contractility

Sarcomere shortening was recorded using a video-based edge-detecting IonOptix system (Milton, MA). Only the rod-shaped AMCMs with clear edges and without obvious sarcolemmal blebs or spontaneous contractions fulfilled the selection criteria for experiments. Briefly, AMCMs were loaded into the recording chamber mounted on the stage of an inverted microscope (Nikon, Eclipse Ti-s) and perfused with tyrode solution. The cells were field stimulated at a frequency of 0.5 Hz, and sarcomere length shortening was simultaneously recorded. The contraction amplitude was calculated by deduction of systolic from diastolic sarcomere length and normalized as the percentage of shortening of cell length. At least 15 individual cells from three mice in each group were used for analysis. The contractility of NRCMs was measured using the Maestro Pro multi-well MEA platform (Axion BioSystems) as previously described [57]. The amplitude of contraction was used as a parameter for measuring contractile change in beating. Independent experiments were repeated at least three times with at least three replicates.

### Western blot and Immunoprecipitation

Cells or tissues were solubilized at 4 °C for 30 min in RIPA lysis buffer (Beyotime, P0013C) containing protease inhibitor (Roche, 4906845001) and phosphatase inhibitor mixture (Roche, 4693116001). The lysates were then cleared by centrifugation at 12,000 × *g* for 10 min at 4 °C. For immunoprecipitation, the supernatants were incubated with anti-PKM2 antibody or IgG overnight at 4 °C, and the immune complexes were captured by protein A/G agarose (Beyotime, P2012) for 3–4 h at 4 °C. Immunoprecipitation or Western blot samples were then denatured and separated by 10% Bis-Tris gel (Invitrogen, NP0315BOX), transferred onto PVDF membranes (Millipore, IPVH00010), and incubated with the corresponding primary antibodies. The primary antibodies were listed in Table S3. The images of blots were captured with an Odyssey imager (LI-COR, Biosciences), and band intensity was quantified using open-source ImageJ software.

### RNA purification and quantitative real-time polymerase chain reaction (qRT-PCR)

RNA purification and qRT-PCR were performed as described in our previous study [58]. Briefly, total RNA purification from neonatal and adult cardiomyocytes or heart tissue was conducted with RNAiso plus reagent (Takara, 9109) based on the manufacturer’s protocol. 1 μg of total RNA from each sample was used to generate cDNA with the PrimeScript^TM^ RT reagent Kit (Takara, RR037A). qRT-PCR was performed using SYBR Green PCR Master Mix (Toyobo, QPK-201) on QuantStudio 6 real-time polymerase chain reaction system (Applied Biosystems). The *18S* and *Gapdh* genes were utilized as an internal control. The relative values were determined by the 2^−ΔΔCT^ algorithm and expressed as the fold change normalized to the expression of internal controls. Specific primers used were listed in Table S4.

### Histology analysis

After LPS stimulation for 24 h, mice were euthanized, and then the heart was dissected for histological analysis with paraffin imbedded sections. The 6 μm thick sections were used for morphological analysis. Hematoxylin-eosin (HE) staining was performed according to the manufacturer’s protocol of the HE staining kit (Beyotime, C0105S). For immunohistochemistry, sections were heated in citrate buffer to facilitate antigen retrieval and then incubated with an anti-CD68 antibody (Proteintech, 28058-1-AP) or TUNEL probe (Roche, 12156792910). Secondary antibodies used in this study included Alexa Fluor 488 conjugated goat anti-mouse and Alexa Fluor 555 conjugated goat anti-rabbit. The percentage of cells shown to be TUNEL or CD68 positive was counted by ImageJ 2.1 software.

### Immunofluorescence

Immunofluorescence was performed in accordance with the previous procedure with slight modifications [59]. Concisely, cells were seeded into a glass-bottom cell culture dish at a proper cell density. Then cells were fixed for 15 min with 4% (wt/vol) paraformaldehyde (Sigma, p1648) in PBS, followed by permeabilization with PBS containing 0.5% Triton X-100 (PBST) for 13 min. After blocking with 4% goat serum (Gibco, 16210072) in PBST, cells were incubated with primary antibodies (PKM2, SERCA2a, Flag, GFP, etc.) overnight at 4 °C and incubated with Alexa Fluor conjugated secondary antibodies. Next, nuclei were stained with DAPI (Sigma, D9542) for 5 min. Images were captured by a laser confocal microscopy, and were quantified by Image J software.

### HEK-293T cell culture and transfection

HEK-293T cells were cultured in high glucose Dulbecco’s modified eagle medium (DMEM Gibco, C11995500BT) containing 10% fetal bovine serum (FBS, Excell, FND500) at 37 °C, 5% CO_2_. When the cells reached 60–70% confluence, they were co-transfected with Flag-SERCA2a, and PKM2 or its truncated forms (*Pkm2* full-length, domain of exon 10 and *Pkm2*-Δexon 10) fused with GFP-tag using Lipofectamine^TM^ 3000. Co-immunoprecipitation or immunofluorescence microscopy was processed at 48 h post-transfection.

### Statistical analysis

All data were represented as mean ± SEM (standard error of the mean) from at least three individual experiments. A *p* value of <0.05 was considered to represent a significant difference. Statistical analyses were performed using GraphPad Prism software 8.0 (GraphPad Software Inc). Data have been analyzed for normality and equal variance as a pre-condition or justification for using Student’s t-test and one-way ANOVA. When appropriate, unpaired or paired two-tailed Student’s *t*-test, one-way analysis of variance (ANOVA), and two-way ANOVA were carried out to determine the statistical significance of the data.

## Supplementary information

Supplementary Material

Original Data File

Agreement of Changes to authorship

## Data Availability

All relevant data are included in the article and Supplementary materials. The additional information related to this paper is available from the corresponding author on reasonable request.
