# Supplementary material for: PKM2 deficiency exacerbates gram-negative sepsis-induced cardiomyopathy via disrupting cardiac calcium homeostasis
Source: Cell Death Discov. 2022 Dec 23;8:496. doi: 10.1038/s41420-022-01287-9 (PMC9789059; doi:10.1038/s41420-022-01287-9)
Supplement: Supplementary file 1 — Supplementary Material [file 41420_2022_1287_MOESM1_ESM.docx]

**Supplemental Material**

**
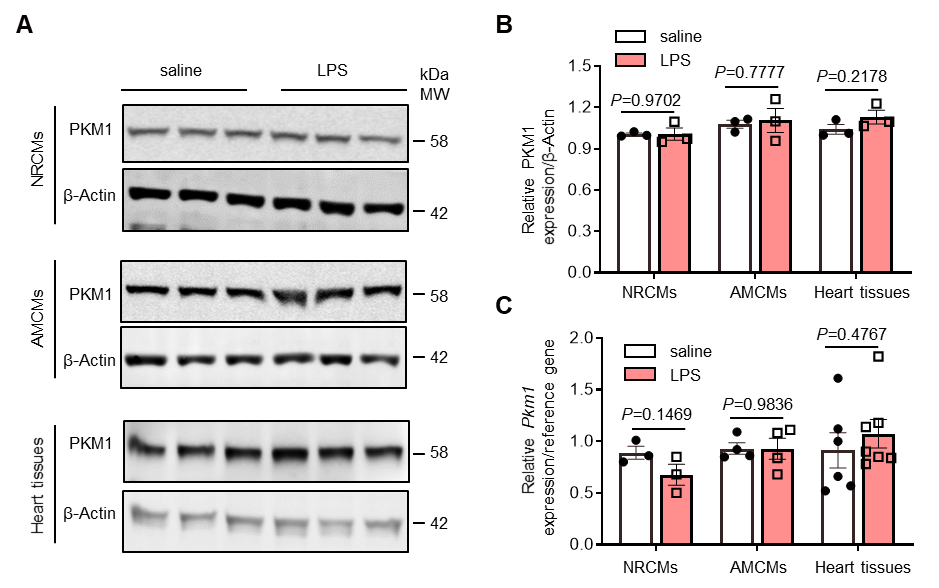
**

**Fig. S1: PKM1 remains unchanged in cardiomyocytes in response to lipopolysaccharide (LPS). A** Representative image of Western blot of PKM1 expression in neonatal rat cardiomyocytes (NRCMs), adult mouse cardiomyocytes (AMCMs) and heart tissues with saline or LPS administration for 24 h. β-Actin served as loading control. **B** Quantitation of PKM1 expression in NRCMs, AMCMs and heart tissues with saline or LPS administration for 24 h, n = 3. **C** Quantitative real-time polymerase chain reaction (qRT-PCR) analysis of *Pkm1* in NRCMs, AMVMs and heart tissues administrated with LPS. *18S* was used as an internal reference gene in NRCMs. *Gapdh* was used as an internal reference gene in AMVMs and heart tissues, n = 3 to 7. Values represent the mean ± SEM of at least three independent experiments.

**
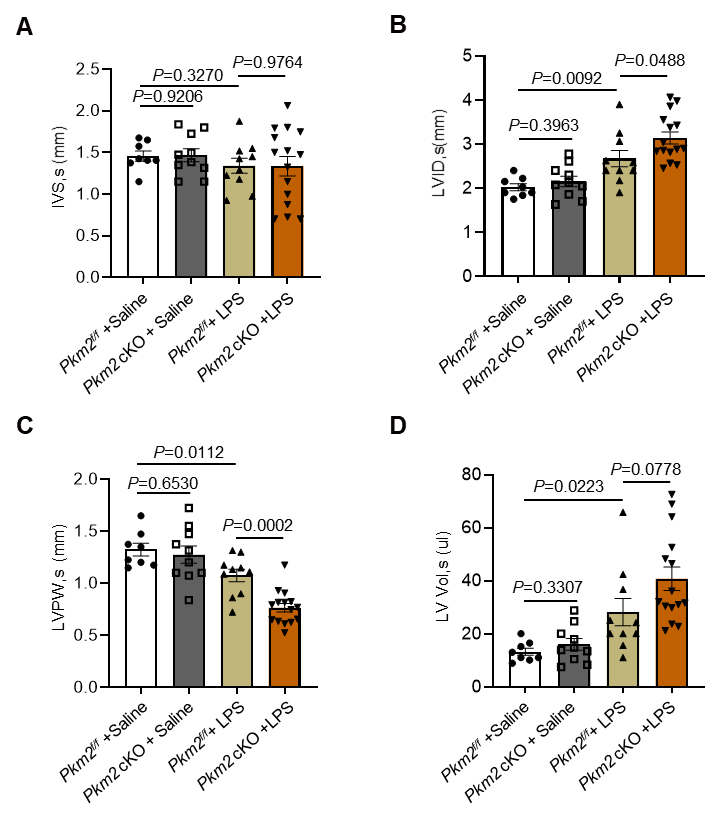
**

**Fig. S2: Cardiomyocyte-specific *Pkm2* knockout exacerbated LPS**-**induced systolic dysfunction. A** Systolic interventricular septum (IVS, s), **B** left ventricular internal systolic (LVID, s), **C** left ventricular posterior wall (LVPW, s) and **D** left ventricular volume (LV vol, s) evaluated by echocardiography in *Pkm2^f/f^* and *Pkm2* cKO mice after saline or LPS administration. n = 8 to 15. Values represent the mean ± SEM.


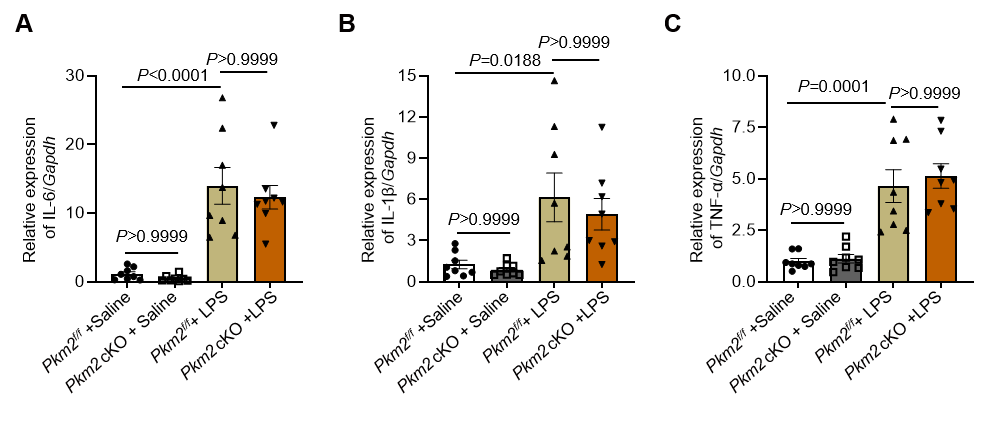


**Fig. S3: Cardiomyocyte-specific *Pkm2* knockout rarely affected heart inflammation after LPS stimulation. A-C** Quantitative real-time polymerase chain reaction (qRT-PCR) analysis of IL-6, TNF-α and IL-1β in heart tissues from *Pkm2* cKO and *Pkm2^f/f^* mice administrated with LPS. *Gapdh* was used as an internal reference gene, n = 8. Values represent the mean ± SEM of at least three independent experiments.

**
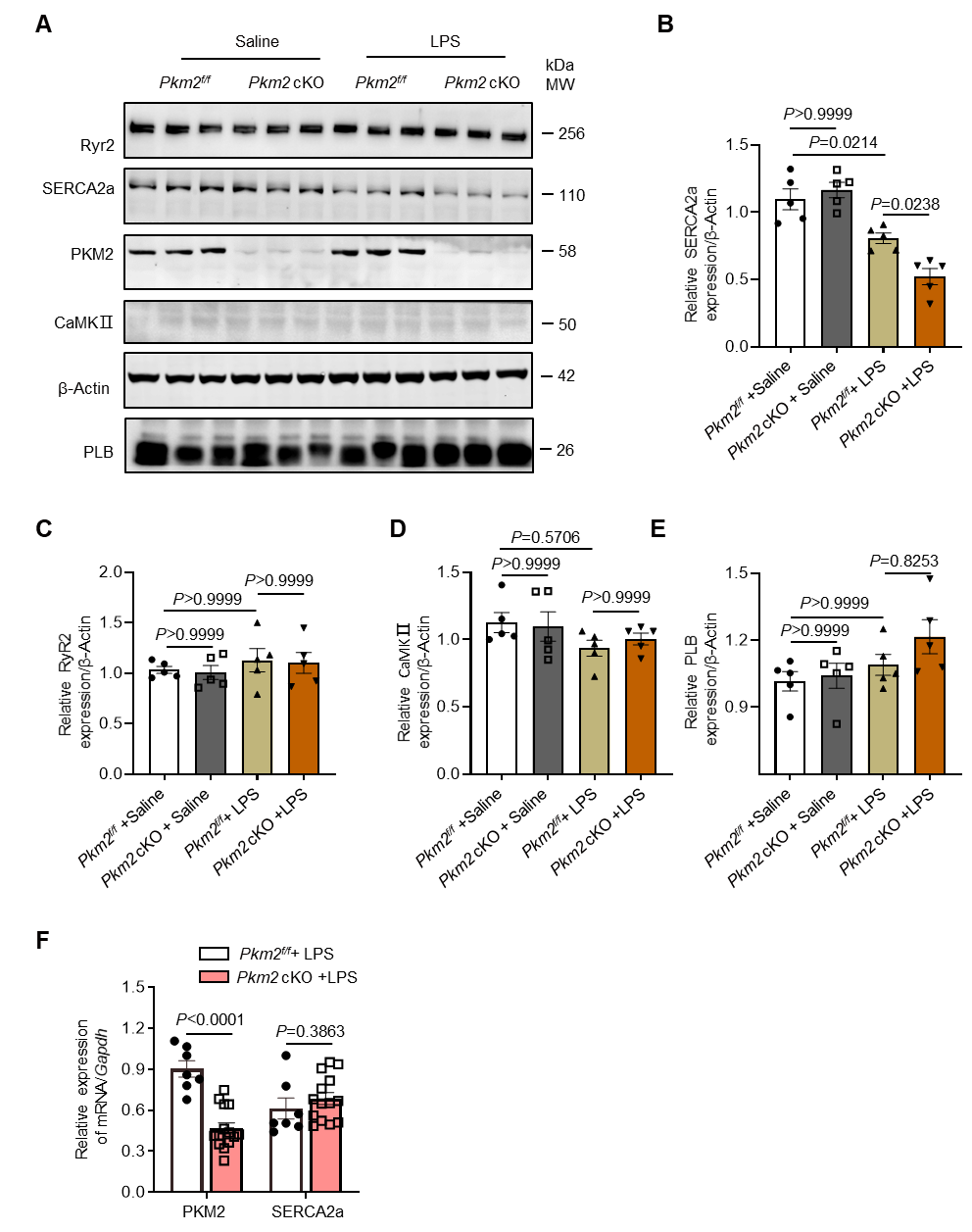
**

**Fig. S4: PKM2, rather than PKM1, regulated SERCA2a. A**-**E** Western blot and quantitation of PKM2, PLB, RyR2, CaMKII and SERCA2a expression in hearts from *Pkm2* cKO and *Pkm2^f/f^* mice 24 h after saline or LPS administration. β-Actin served as loading control, n = 5. **F** Quantitative real-time polymerase chain reaction (qRT-PCR) analysis of *Pkm2* and *Serca2a* in heart tissues from *Pkm2* cKO and *Pkm2^f/f^* mice administrated with LPS. *Gapdh* was used as an internal reference gene, n = 7 to 14. Values represent the mean ± SEM of at least three independent experiments.


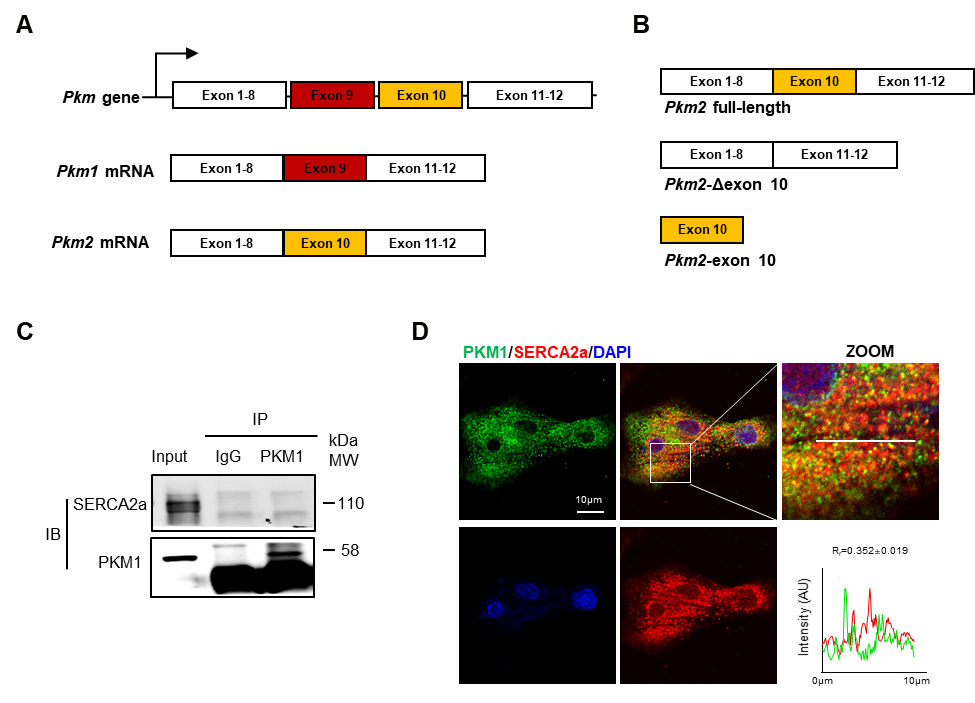


**Fig. S5: PKM1 did not interact with SERCA2a. A** Schematic diagram showing the PKM gene alterative splicing products. **B** Illustration of *Pkm2* full-length, truncated mutant (*Pkm2*-Δexon 10) and exon 10. **C** Immunoprecipitation assay validated no direct interaction between SERCA2a and PKM1 with anti-PKM1 antibody in NRCMs. **D** Representative immunofluorescence images showing distinct localization of PKM1 (green) and SERCA2a (red) in NRCMs by confocal immunofluorescence analysis (scale bars, 10 μm). Line profile analyses showing the distribution and intensity of fluorescence signals. The Pearson’s coefficient was measured from the images using ImageJ software (n = 15). Values represent the mean ± SEM.


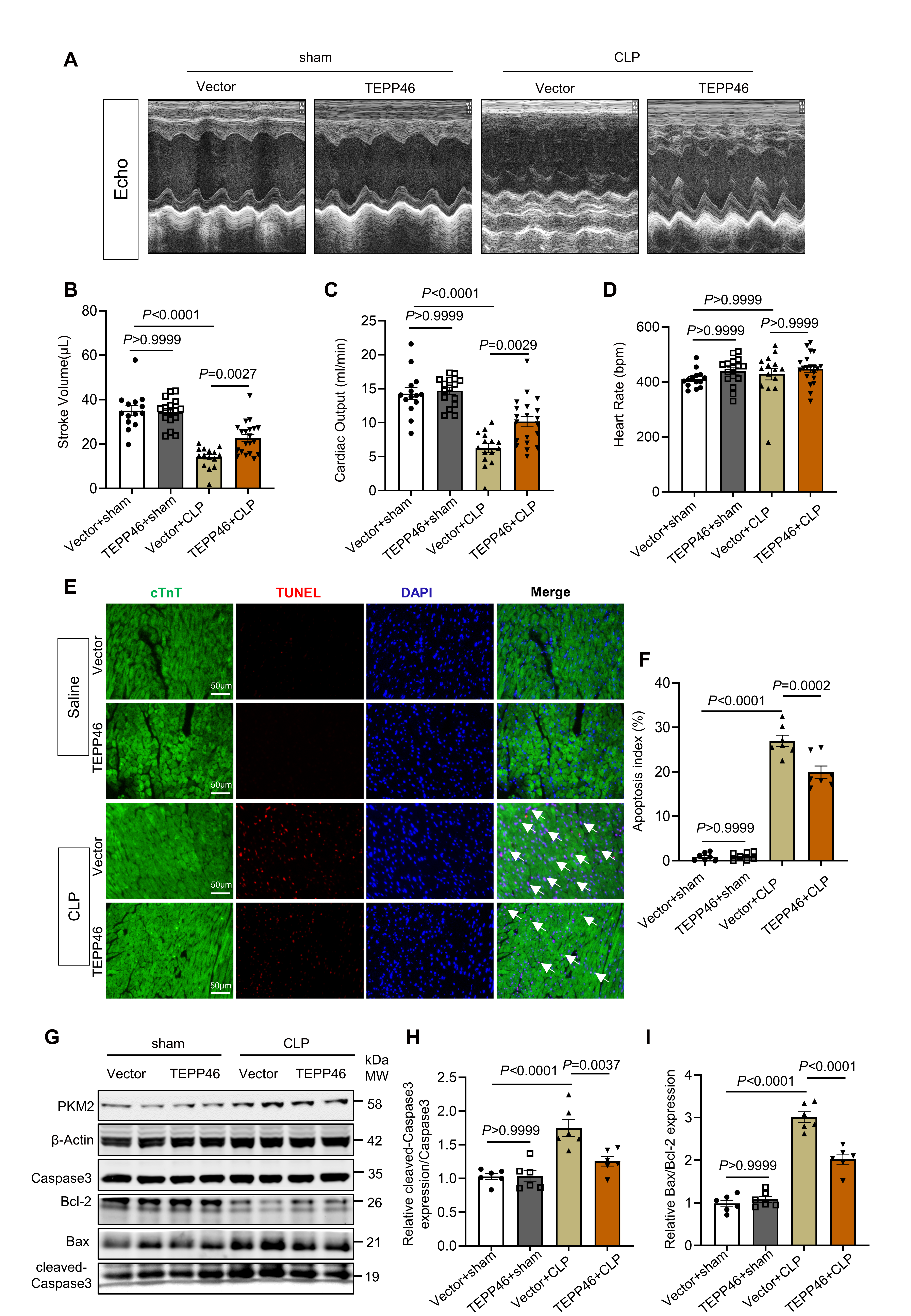
**Fig. S6: TEPP46 alleviated cecum ligation and puncture (CLP)-induced cardiomyopathy in vivo. A** Representative M-mode echocardiography from mice after CLP surgery co-treated with saline or TEPP46. **B-D** Quantitative analysis of stroke volume (SV), cardiac output (CO), and heart rate by echocardiography, n = 14 to 20. **E, F** Representative images and quantification of TUNEL staining, n = 7. Scale bar: 50 µm. White arrows point to TUNEL positive cardiomyocytes. **G-I** Western blot and quantitation of apoptosis markers (cleaved-Caspase 3/Caspase3 and Bax/Bcl-2 ratio) expression in hearts of mice after CLP surgery co-treated with saline or TEPP46. β-Actin served as the loading control, n = 6.

**
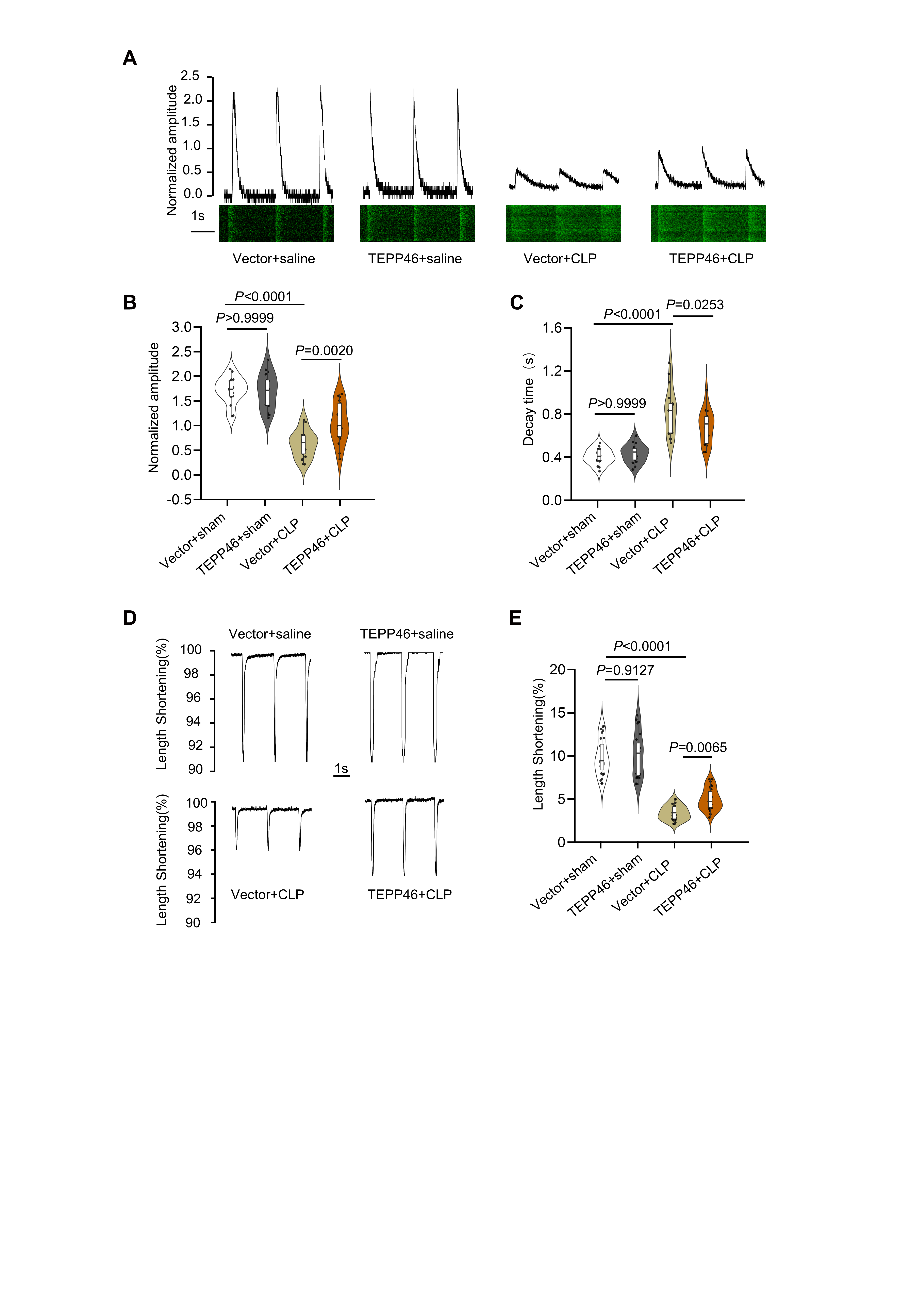
**

**Fig. S7: TEPP46 protected cardiomyocytes from CLP-induced systolic disfunction. A-C** Representative calcium transient curve and quantitation of calcium transient amplitudes and decay time in AMCMs from mice after CLP surgery co-treated with saline or TEPP46, n = 17. **D, E** Cardiomyocytes contraction depicting cell shortening in cardiomyocytes isolated from mice after CLP surgery co-treated with saline or TEPP46, n = 28. Values represent the mean ± SEM.

**Table S1. The primer sequences for genotyping.**

| Primer | Primer sequences |
| --- | --- |
| mouse-cre-F | TCTATTGCACACAGCAATCCA |
| mouse*-cre*-R | CCAGCATTGTGAGAACAAGG |
| mouse*-Pkm2^f/f^*-F | CCTTCAGGAAGACAGCCAAG |
| mouse*-Pkm2^f/f^*-R | AGTGCTGCCTGGAATCCTCT |

**Table S2. The siRNA sequences.**

| Sequence names | Sequences |
| --- | --- |
| Rat-si-*Pkm2*-2-F | CCCAAGGGCUCCUAUCAUU |
| Rat-si-*Pkm2*-2-R | AAUGAUAGGAGCCCUUGGG |
| NC-F | GGUUGUGCAAGAGGGCUUU |
| NC-R | AAAGCCCUCUUGCACAACC |

**Table S3. Antibodies used in this study.**

| Antibody | Application | Source | Cat No |
| --- | --- | --- | --- |
| Bax | WB | Proteintech | 50599-2-Ig |
| Bcl2 | WB | Proteintech | 26593-1-AP |
| CaMKII | WB | Abcam | ab181052 |
| Caspase3 | WB | Proteintech | 19677-1-AP |
| CD68 | IF | Proteintech | 28058-1-AP |
| Flag | WB/IF | Sigma-aldrich | F1804 |
| GFP | WB/IF | Sino Biological | 13105-R208 |
| PKM1 | WB/IF/IP | Proteintech | 15821-1-AP |
| PKM2 | WB | Proteintech | 60268-1-Ig |
| PKM2 | WB/IF/IP | Cell Signaling Technology | 4053 |
| PLB | WB | Cell Signaling Technology | 14562 |
| RyR2 | WB | Proteintech | 19765-1-AP |
| SERCA2a | WB/IF | Novus | NB300-581 |
| β-Actin | WB | Santa Cruz | sc-47778 |

WB, western blot; IF, immunofluorescence; IP, immunoprecipitation.

**Table S4. The primer sequences for qPCR analysis.**

| Primer | Primer sequences |
| --- | --- |
| mouse-*Pkm1*-F | AGCCTCCAGTCACTCCACAGA |
| mouse-*Pkm1*-R | TCAGCACGGCATCCTTACAC |
| rat-*Pkm1*-F | AGCCTCCAGTCAATCCACAGA |
| rat-*Pkm1*-R | ACGGCATCCTTACACAGCACA |
| mouse/rat-*Pkm2*-F | ATTACCAGCGACCCCACAGAA |
| mouse/rat-*Pkm2*-R | ACGGCATCCTTACACAGCACA |
| mouse-*Gapdh*-F | CCAGCTACTCGCGGCTTTA |
| mouse-*Gapdh*-R | CCAATACGGCCAAATCCGTTC |
| mouse-*Serca2a*-F | TGGAACCTTTGCCGCTCATT |
| mouse-*Serca2a*-R | TTCCCCAACCTCAGTCATGC |
| rat-*18S*-F | GTTGAACCCCATTCGTGAT |
| rat-*18S*-R | GCTTATGACCCGCACTTACT |
| mouse-TNF-α-F | AACCTCCTCTCTGCCGTCAA |
| mouse-TNF-α-R | AAAGTAGACCTGCCCGGACTC |
| mouse-IL-6-F | TGGAGTCACAGAAGGAGTGGCTAA |
| mouse-IL-6-R | TCTGACCACAGTGAGGAATGTCCA |
| mouse-IL-1β-F | TGCCACCTTTTGACAGTGATG |
| mouse-IL-1β-R | TGTGCTGCTGCGAGATTTGA |
