# Supplementary material for: PKM2 deficiency exacerbates gram-negative sepsis-induced cardiomyopathy via disrupting cardiac calcium homeostasis
Source: Cell Death Discov. 2022 Dec 23;8:496. doi: 10.1038/s41420-022-01287-9 (PMC9789059; doi:10.1038/s41420-022-01287-9)

Figure1 A

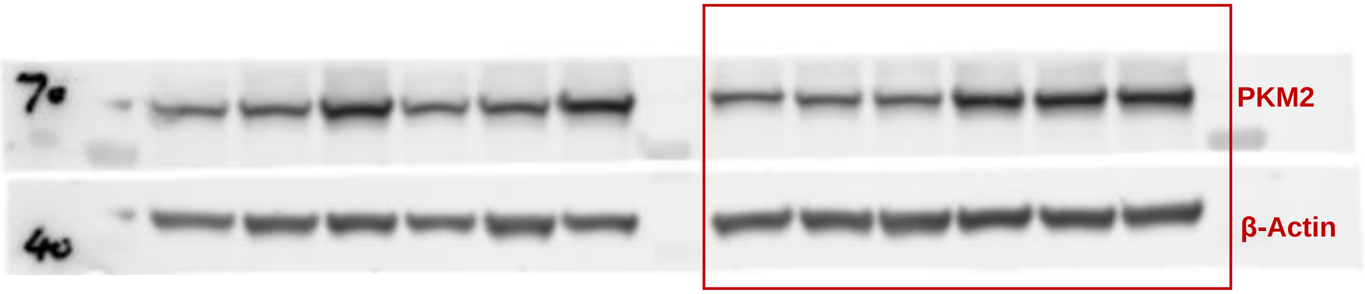

Figure1 B

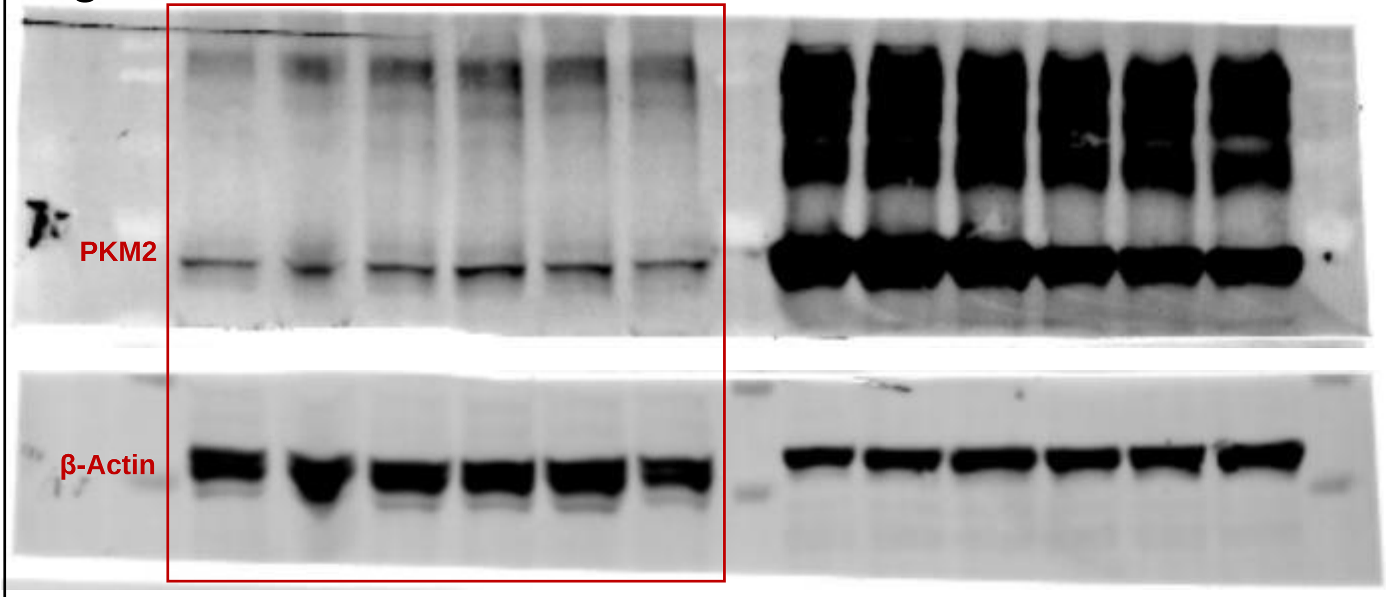

Figure1
C

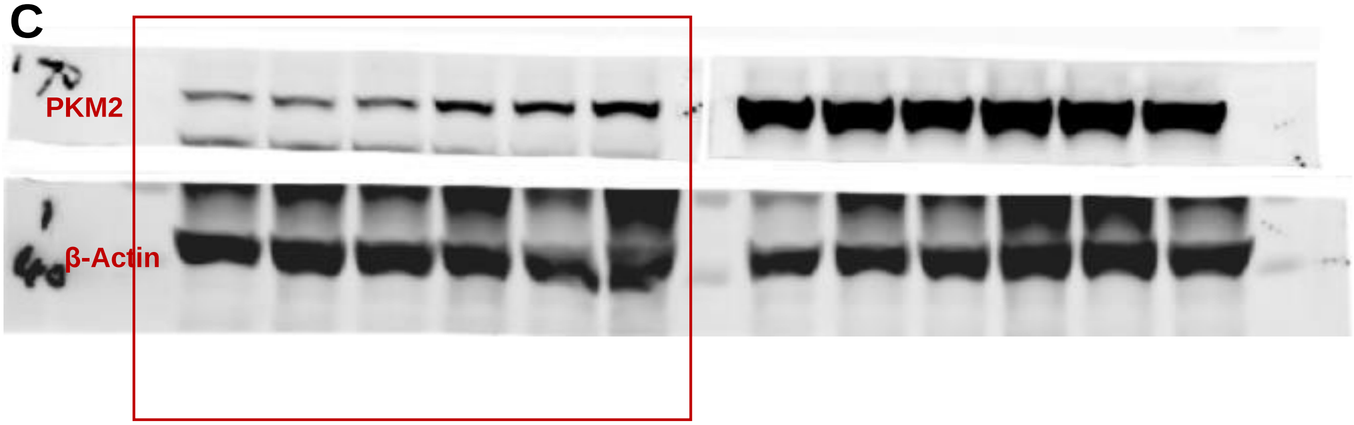

Figure2 A

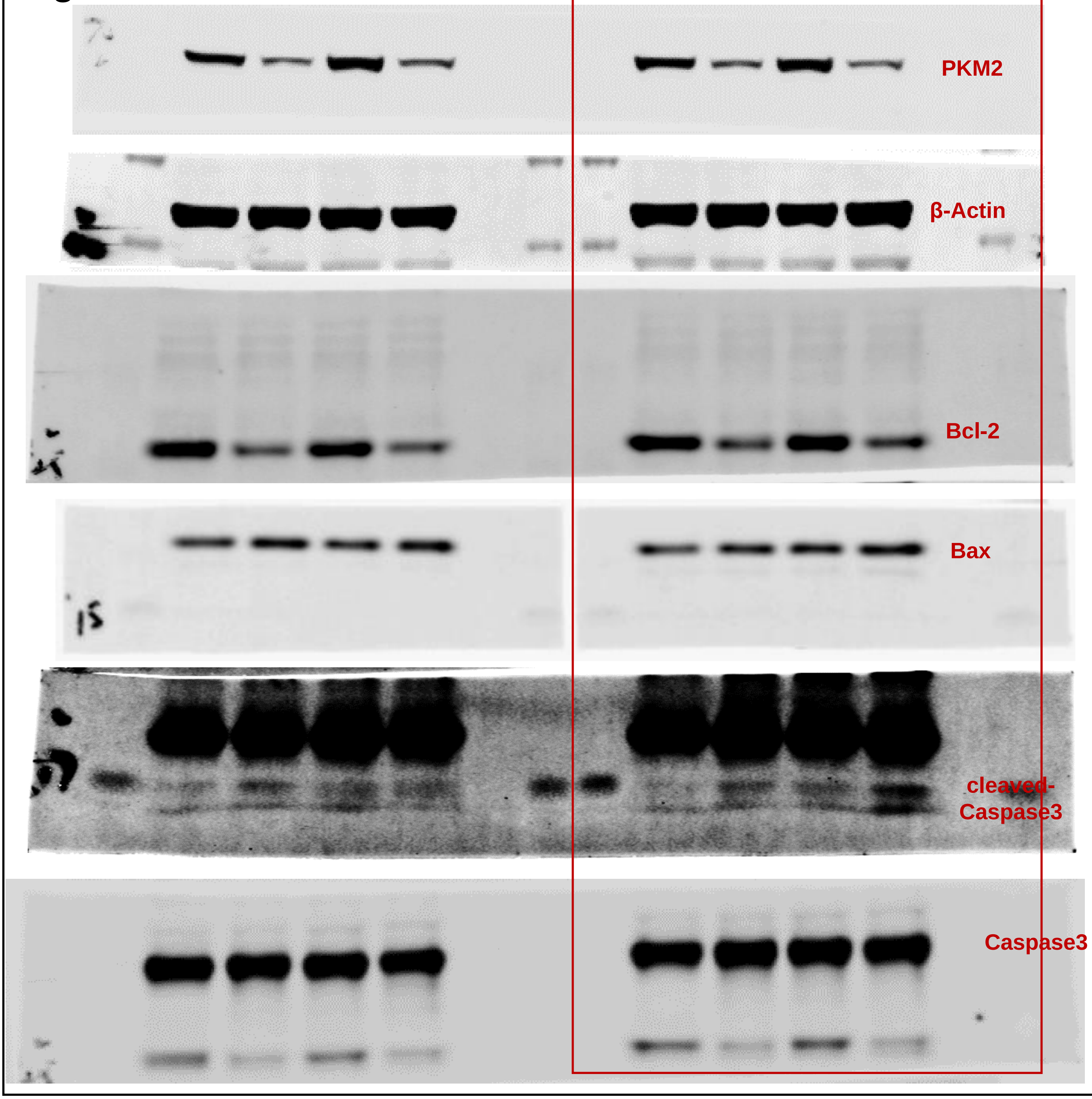

Figure3 H

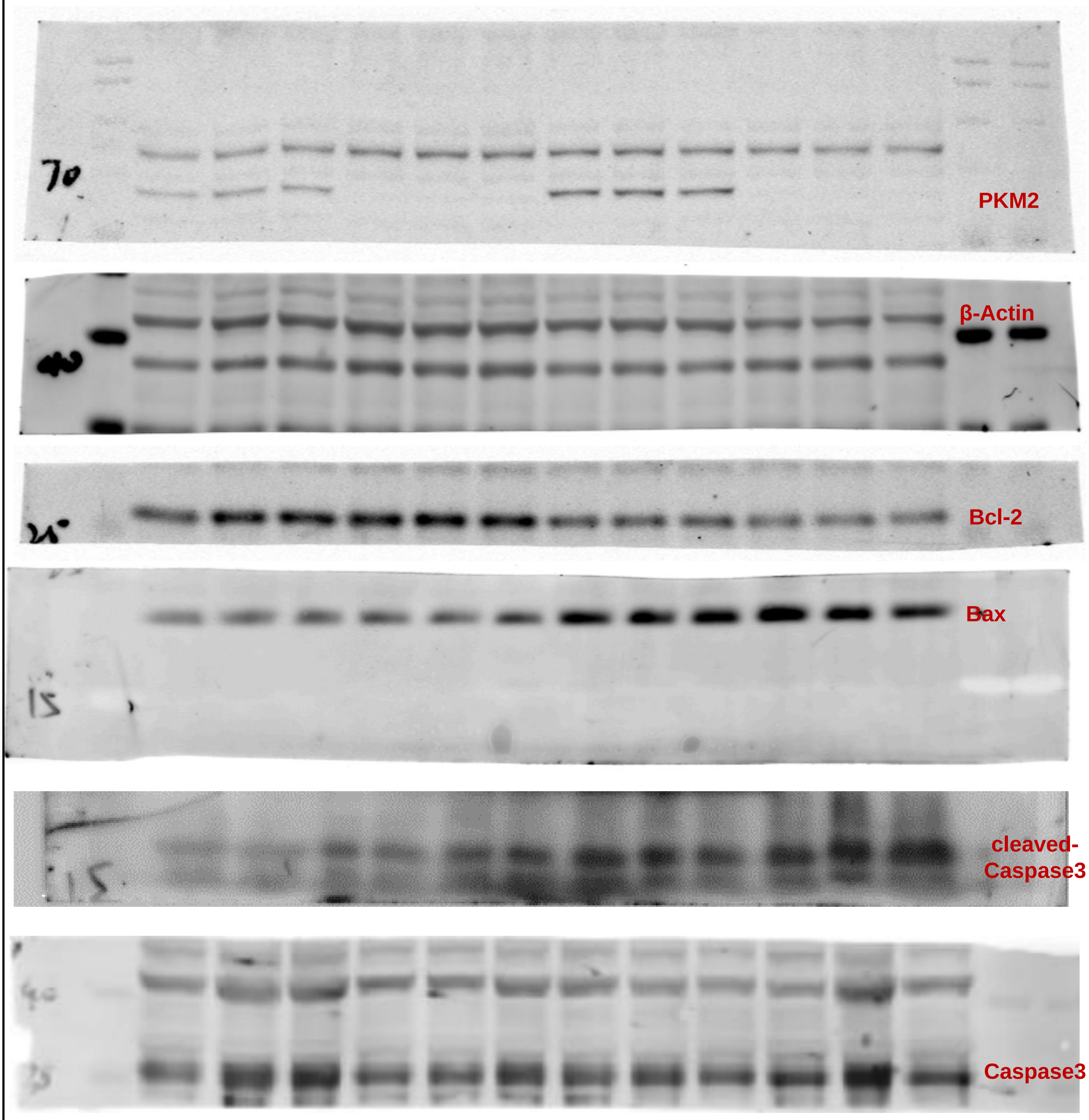

70
PKM2
β-Actin
40
Bcl-2
25
Bax
15
cleaved-Caspase3
15
Caspase3

Figure4 A

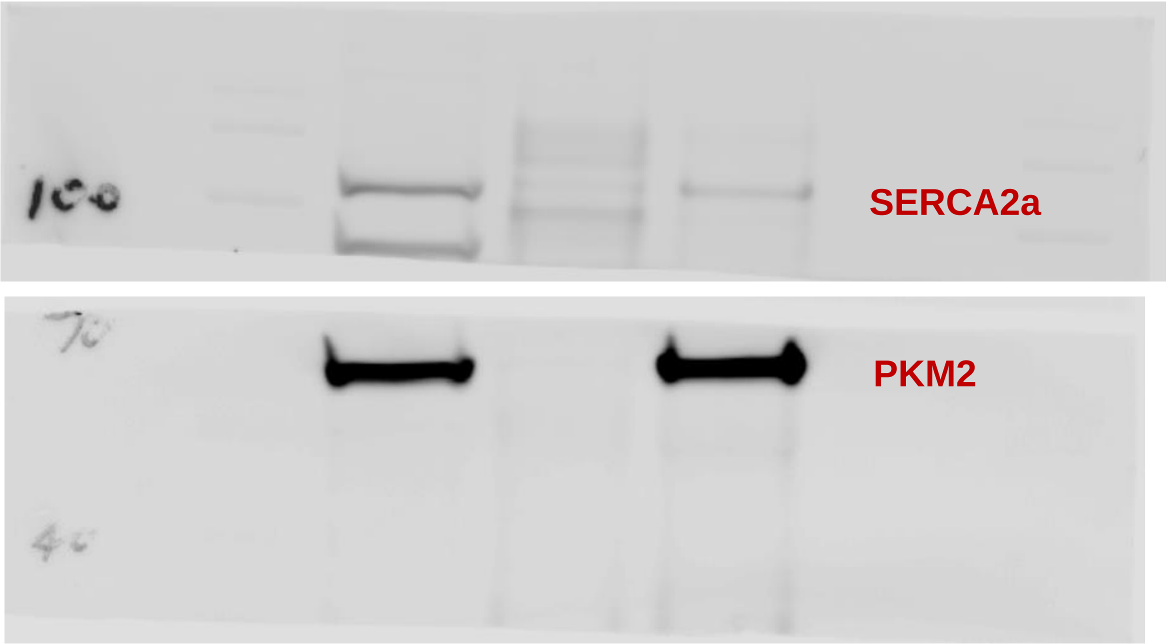

Figure4 D

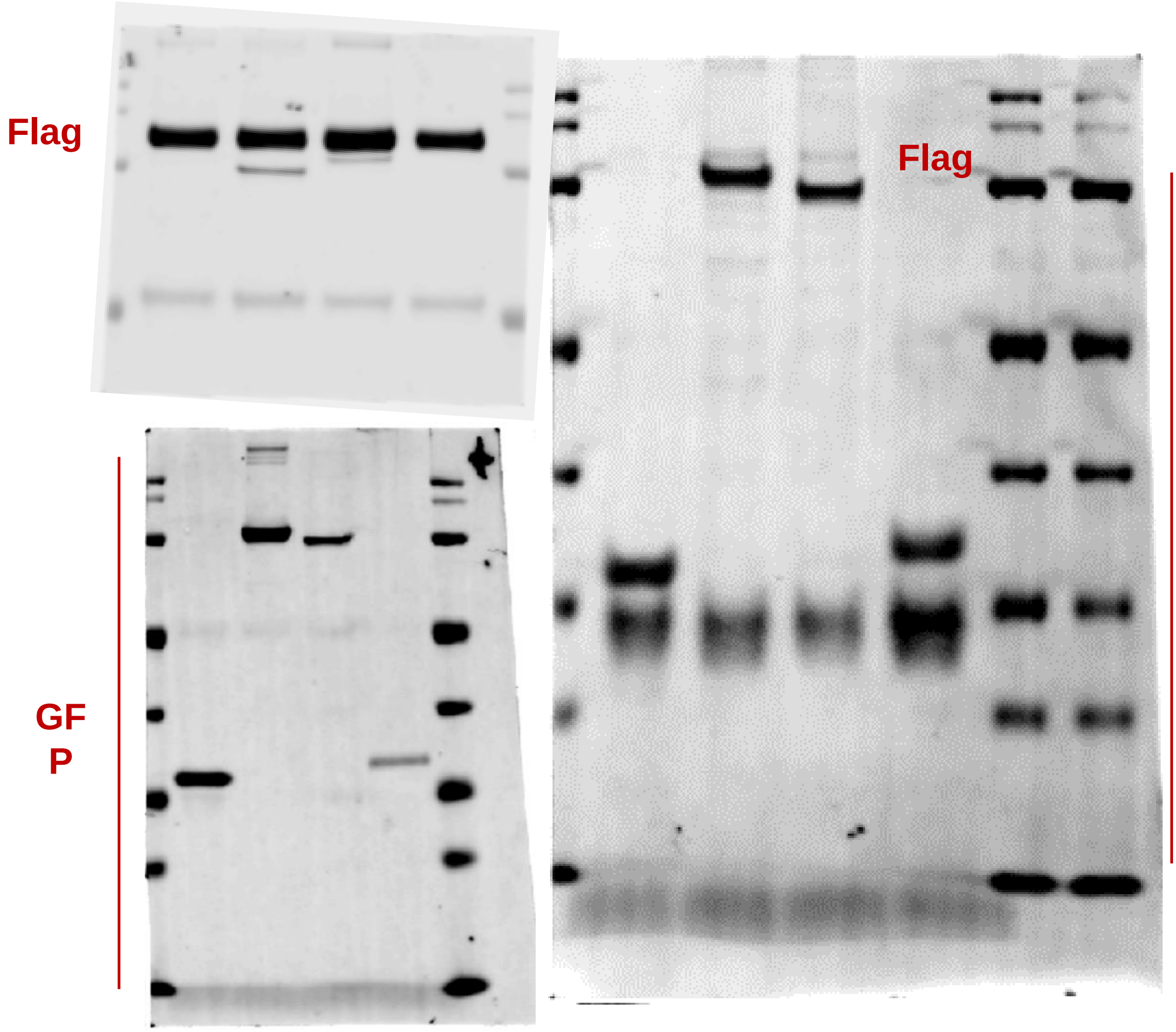

Figure4 E

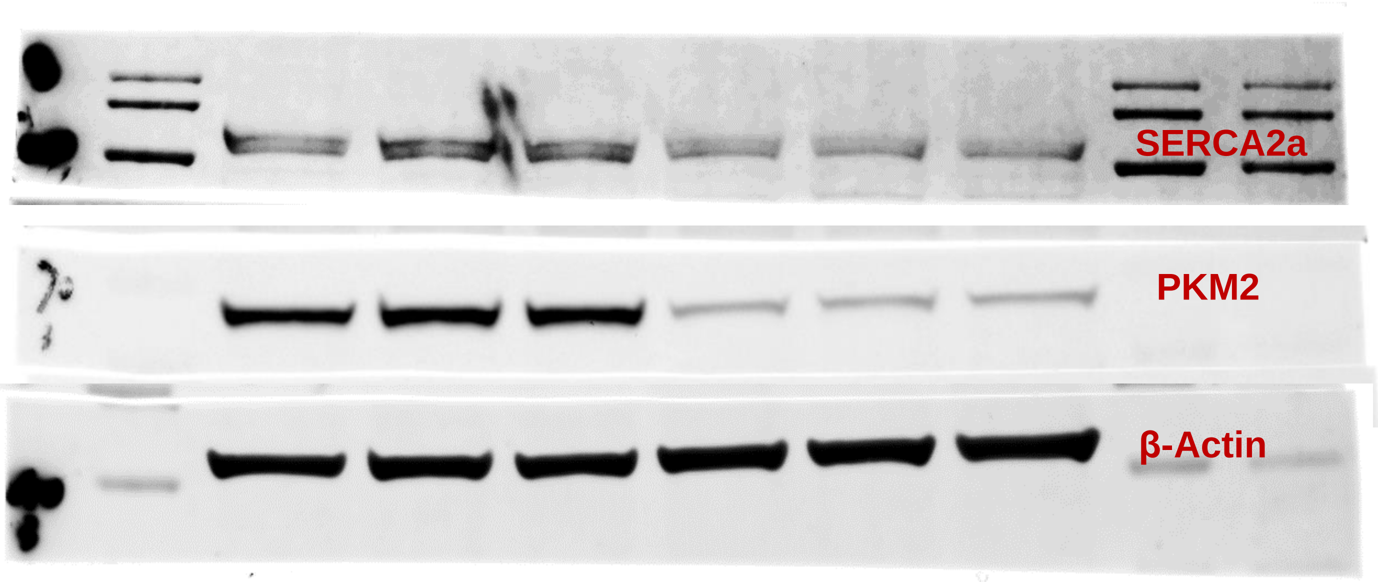

Figure4 G

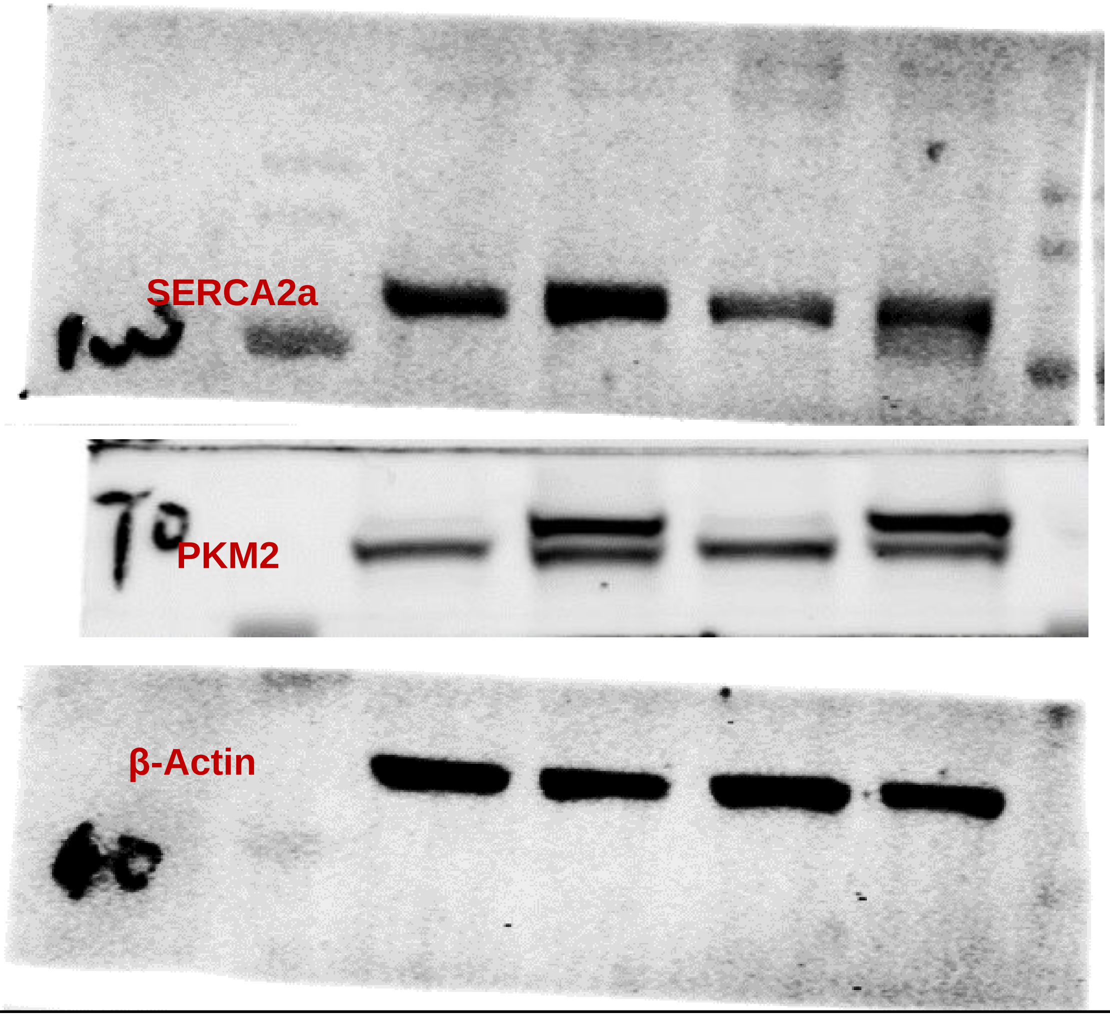

Figure5 A

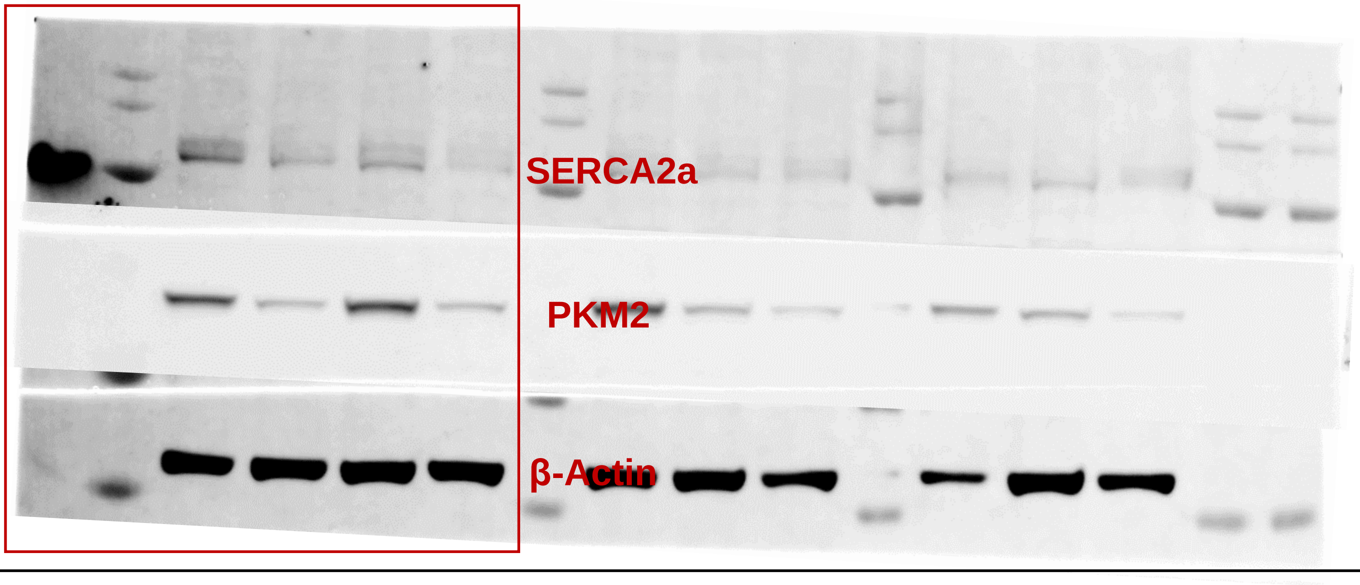

Fig. S1A NRCMs

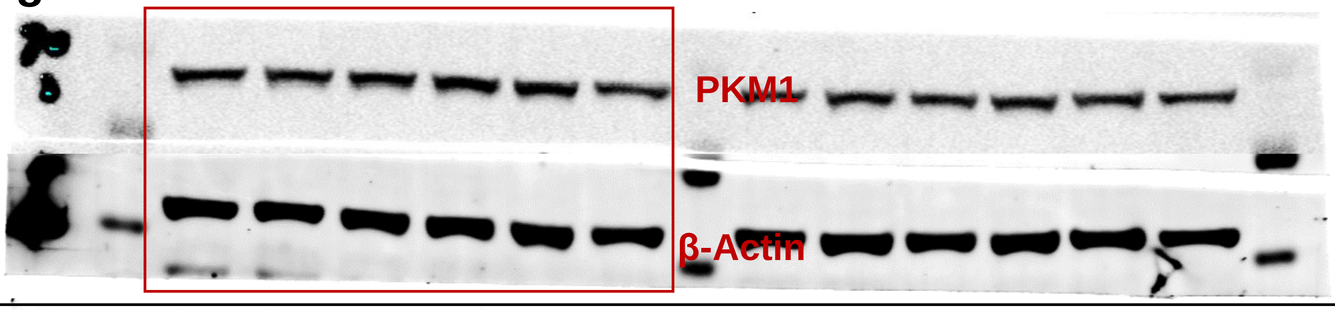

Fig. S1A AMVMs

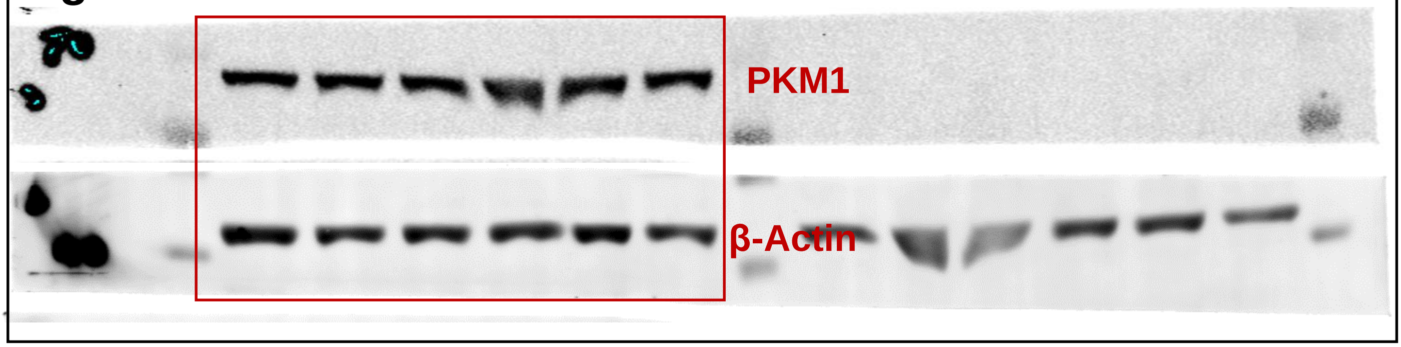

Fig. S1A heart tissues

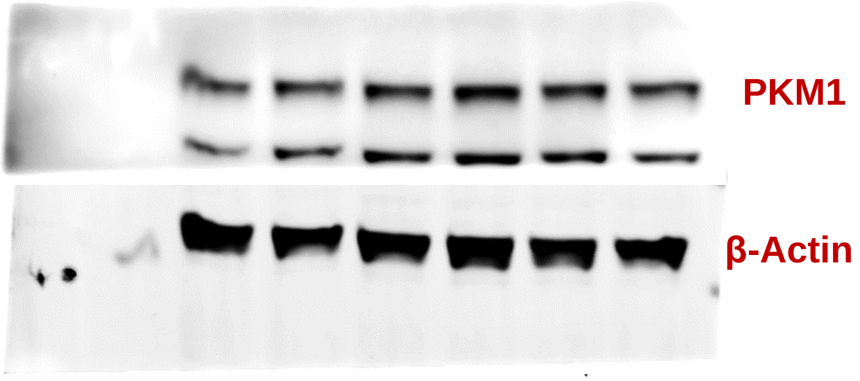

Fig. S3A

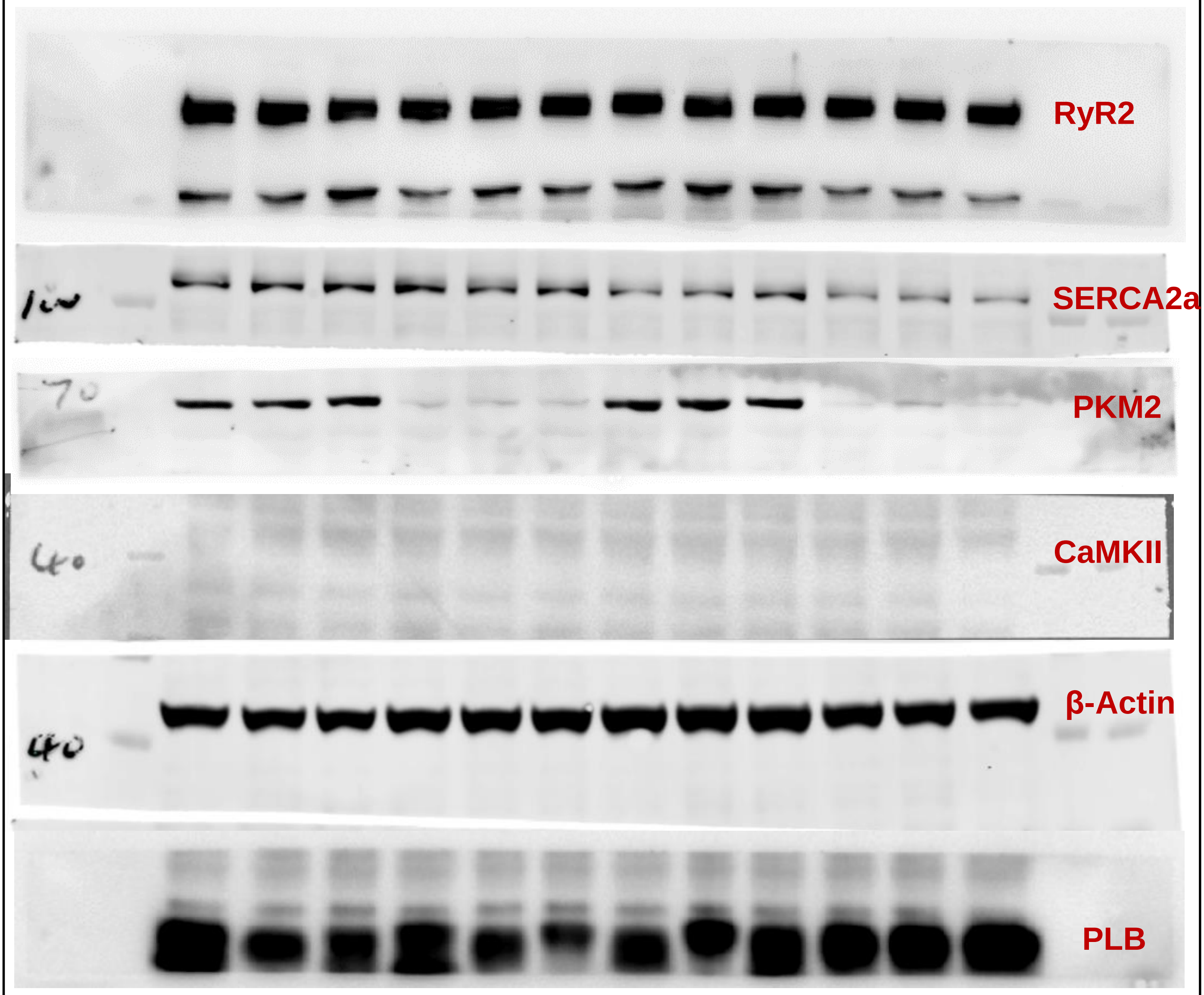

Fig. S3G

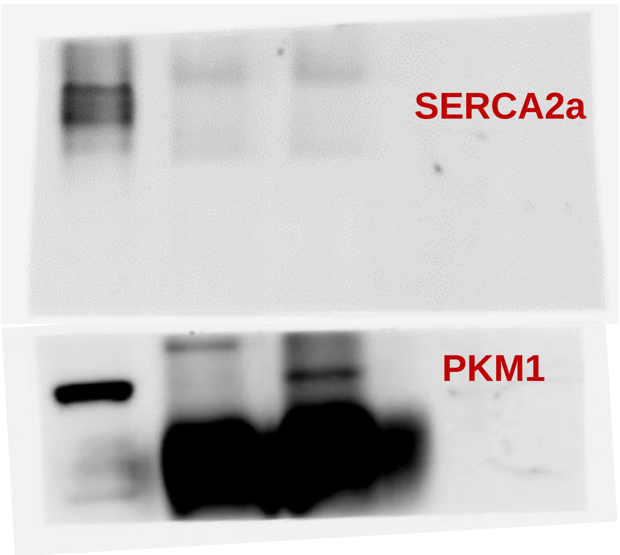

Fig. 7G

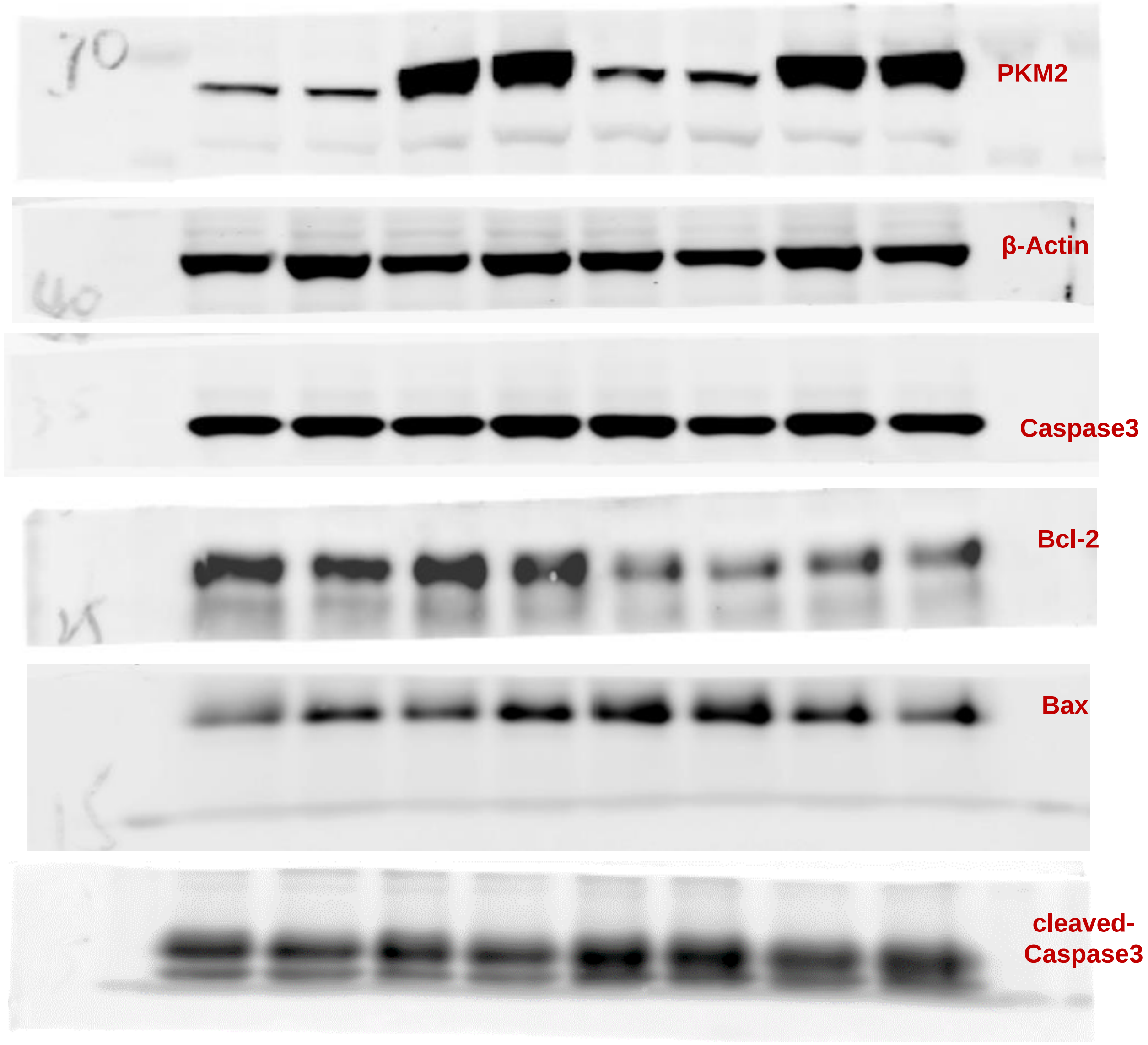

Fig. S6G

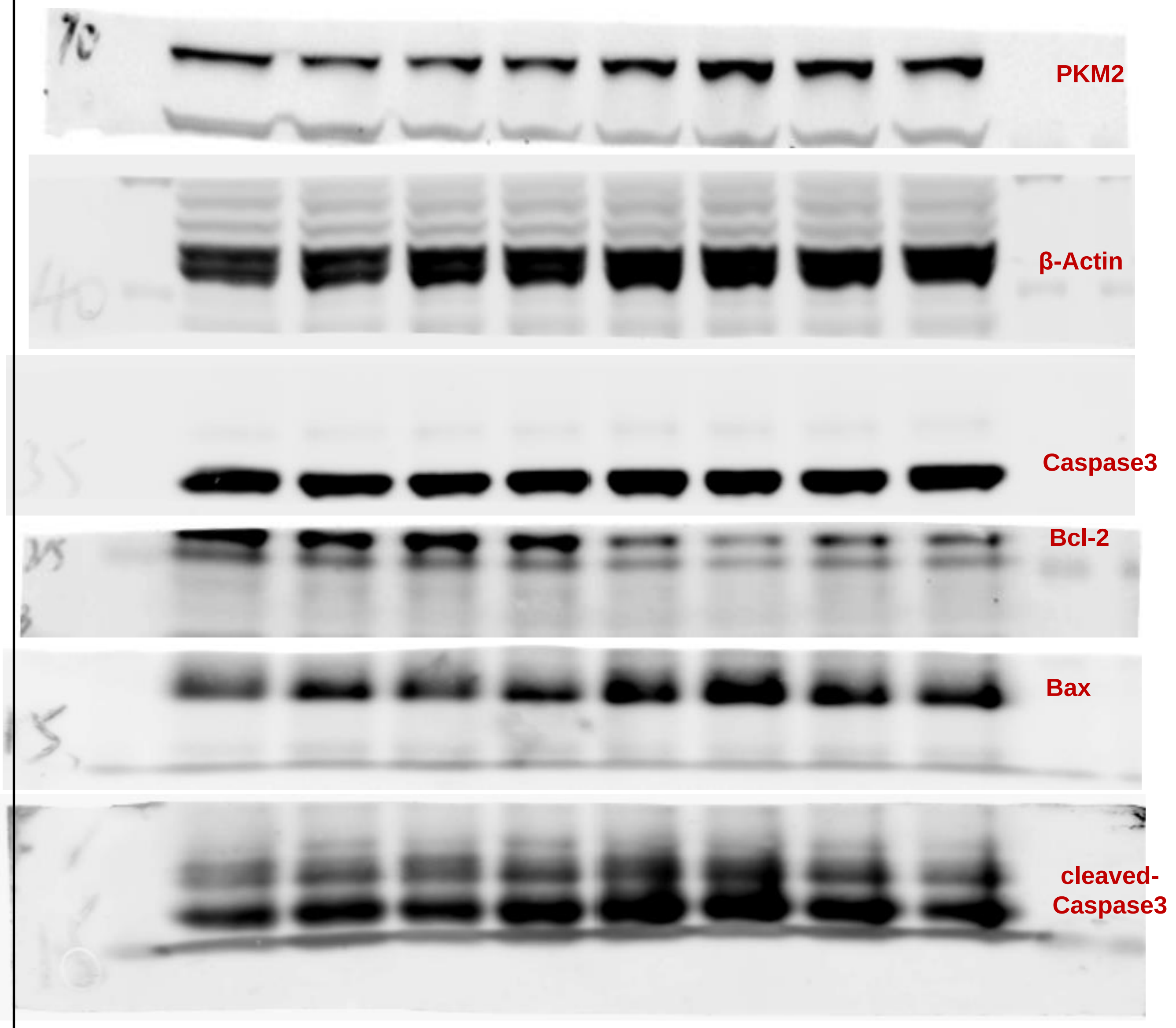

Supplement: Supplementary file 2 — Original Data File [file 41420_2022_1287_MOESM2_ESM.pdf]
