# Supplementary material for: PKM2 deficiency exacerbates gram-negative sepsis-induced cardiomyopathy via disrupting cardiac calcium homeostasis
Source: Cell Death Discov. 2022 Dec 23;8:496. doi: 10.1038/s41420-022-01287-9 (PMC9789059; doi:10.1038/s41420-022-01287-9)
Supplement: Supplementary file 3 — Agreement of Changes to authorship [file 41420_2022_1287_MOESM3_ESM.pdf]

发件人: **施丹** shidan@tongji.edu.cn
主题: Changes to authorship
日期: 2022年12月14日 下午12:13
收件人: leni-dfyy leni-dfyy@tongji.edu.cn, Bowen Lin bowenlin0512@163.com, shenmeiting@tongji.edu.cn, lican0210@gmail.com, hugeer@tongji.edu.cn, Fu15556667713@163.com, jy279@tongji.edu.cn, 陈 磊 mdchenlei@outlook.com

施

Dear co-authors,
Manuscript Number: CDDISCOVERY-22-4968R1
Title: PKM2 deficiency exacerbates gram-negative sepsis-induced cardiomyopathy via disrupting cardiac calcium homeostasis

Considering that Can Li and Lei Chen have made adequate contributions during the revision, I would like to ask for your agreement to change/add their names in the final author lists.
The raw order of the authors was: Le Ni, Bowen Lin, Meiting Shen, Lingjie Hu, Fengmei Fu, Can Li, Jian Yang, Dan Shi
The final order of the authors was: Le Ni, Bowen Lin, Meiting Shen, Can Li, Lingjie Hu, Fengmei Fu, Lei Chen, Jian Yang, Dan Shi

Please respond “Agree” or "Not agree" to this email.

Yours sincerely,

Dan Shi, MD, PhD
Tongji University School of Medicine

发件人: bowen lin bowenlin0512@163.com
主题: Re: Changes to authorship
日期: 2022年12月14日 下午12:21
收件人: shidan@tongji.edu.cn

BL

Agree

---- Replied Message ----

From	施丹<shidan@tongji.edu.cn>
Date	12/14/2022 12:13
To	leni-dfyy <leni-dfyy@tongji.edu.cn>,<br>Bowen Lin<bowenlin0512@163.com>,<br>shenmeiting<shenmeiting@tongji.edu.cn>,<br>lican0210<lican0210@gmail.com>,<br>hugeer<hugeer@tongji.edu.cn>,<br>Fu15556667713<fu15556667713@163.com>,<br>jy279<jy279@tongji.edu.cn>,<br>陈 磊<mdchenlei@outlook.com>
Subject	Changes to authorship

Dear co-authors,
Manuscript Number: CDDISCOVERY-22-4968R1
Title: PKM2 deficiency exacerbates gram-negative sepsis-induced cardiomyopathy via disrupting cardiac calcium homeostasis

Considering that Can Li and Lei Chen have made adequate contributions during the revision, I would like to ask for your agreement to change/add their names in the final author lists.
The raw order of the authors was: Le Ni, Bowen Lin, Meiting Shen, Lingjie Hu, Fengmei Fu, Can Li, Jian Yang, Dan Shi
The final order of the authors was: Le Ni, Bowen Lin, Meiting Shen, Can Li, Lingjie Hu, Fengmei Fu, Lei Chen, Jian Yang, Dan Shi

Please respond “Agree” or "Not agree" to this email.

Yours sincerely,

Dan Shi, MD, PhD
Tongji University School of Medicine

发件人: jy279@tongji.edu.cn
主题: Re: Changes to authorship
日期: 2022年12月14日 下午12:36
收件人: 施丹 shidan@tongji.edu.cn
抄送: leni-dfyy leni-dfyy@tongji.edu.cn, bowen lin bowenlin0512@163.com, shenmeiting@tongji.edu.cn, lican0210@gmail.com, hugeer@tongji.edu.cn, fu15556667713@163.com, 陈 磊 mdchenlei@outlook.com

J

Dear Dr. Shi,

Thanks for the notification. I agree with the change of authorship.

Best,

Jian Yang Ph.D, Professor

Tongji University School of Medicine

> -----原始邮件-----
> 发件人: "施丹" <shidan@tongji.edu.cn>
> 发送时间: 2022-12-14 12:13:08 (星期三)
> 收件人: leni-dfyy <leni-dfyy@tongji.edu.cn>, "Bowen Lin" <bowenlin0512@163.com>, shenmeiting@tongji.edu.cn, lican0210@gmail.com, hugeer@tongji.edu.cn, Fu15556667713@163.com, jy279@tongji.edu.cn, "陈 磊" <mdchenlei@outlook.com>
> 抄送:
> 主题: Changes to authorship
>
> Dear co-authors,
> Manuscript Number: CDDISCOVERY-22-4968R1
> Title: PKM2 deficiency exacerbates gram-negative sepsis-induced cardiomyopathy via disrupting cardiac calcium homeostasis
>
> Considering that Can Li and Lei Chen have made adequate contributions during the revision, I would like to ask for your agreement to change/add their names in the final author lists.
> The raw order of the authors was: Le Ni, Bowen Lin, Meiting Shen, Lingjie Hu, Fengmei Fu, Can Li, Jian Yang, Dan Shi
> The final order of the authors was: Le Ni, Bowen Lin, Meiting Shen, Can Li, Lingjie Hu, Fengmei Fu, Lei Chen, Jian Yang, Dan Shi
>
> Please respond “Agree” or "Not agree" to this email.
>
> Yours sincerely,
>
> Dan Shi, MD, PhD
> Tongji University School of Medicine

发件人: shenmeiting@tongji.edu.cn
主题: Re: Changes to authorship
日期: 2022年12月14日 下午2:03
收件人: 施丹 shidan@tongji.edu.cn

Agree

> -----原始邮件-----
> 发件人: "施丹" <shidan@tongji.edu.cn>
> 发送时间: 2022-12-14 12:13:08 (星期三)
> 收件人: leni-dfyy <leni-dfyy@tongji.edu.cn>, "Bowen Lin" <bowenlin0512@163.com>, shenmeiting@tongji.edu.cn, lican0210@gmail.com, hugeer@tongji.edu.cn, Fu15556667713@163.com, jy279@tongji.edu.cn, "陈 磊" <mdchenlei@outlook.com>
> 抄送:
> 主题: Changes to authorship
>
> Dear co-authors,
> Manuscript Number: CDDISCOVERY-22-4968R1
> Title: PKM2 deficiency exacerbates gram-negative sepsis-induced cardiomyopathy via disrupting cardiac calcium homeostasis
>
> Considering that Can Li and Lei Chen have made adequate contributions during the revision, I would like to ask for your agreement to change/add their names in the final author lists.
> The raw order of the authors was: Le Ni, Bowen Lin, Meiting Shen, Lingjie Hu, Fengmei Fu, Can Li, Jian Yang, Dan Shi
> The final order of the authors was: Le Ni, Bowen Lin, Meiting Shen, Can Li, Lingjie Hu, Fengmei Fu, Lei Chen, Jian Yang, Dan Shi
>
> Please respond “Agree” or "Not agree" to this email.
>
> Yours sincerely,
>
> Dan Shi, MD, PhD
> Tongji University School of Medicine

发件人: 陈 磊 mdchenlei@outlook.com
主题: 回复: Changes to authorship
日期: 2022年12月14日 下午2:34
收件人: 施丹 shidan@tongji.edu.cn

Agree

发件人: 施丹 <shidan@tongji.edu.cn>
发送时间: 2022年12月13日 20:13
收件人: leni-dfyy <leni-dfyy@tongji.edu.cn>; Bowen Lin <bowenlin0512@163.com>; shenmeiting@tongji.edu.cn <shenmeiting@tongji.edu.cn>; lican0210@gmail.com <lican0210@gmail.com>; hugeer@tongji.edu.cn <hugeer@tongji.edu.cn>; Fu15556667713@163.com <Fu15556667713@163.com>; jy279@tongji.edu.cn <jy279@tongji.edu.cn>; 陈 磊 <mdchenlei@outlook.com>
主题: Changes to authorship

Dear co-authors,
Manuscript Number: CDDISCOVERY-22-4968R1
Title: PKM2 deficiency exacerbates gram-negative sepsis-induced cardiomyopathy via disrupting cardiac calcium homeostasis

Considering that Can Li and Lei Chen have made adequate contributions during the revision, I would like to ask for your agreement to change/add their names in the final author lists.
The raw order of the authors was: Le Ni, Bowen Lin, Meiting Shen, Lingjie Hu, Fengmei Fu, Can Li, Jian Yang, Dan Shi
The final order of the authors was: Le Ni, Bowen Lin, Meiting Shen, Can Li, Lingjie Hu, Fengmei Fu, Lei Chen, Jian Yang, Dan Shi

Please respond “Agree” or "Not agree" to this email.

Yours sincerely,

Dan Shi, MD, PhD
Tongji University School of Medicine

发件人: hugeer@tongji.edu.cn
主题: Re: Changes to authorship
日期: 2022年12月14日 下午4:34
收件人: 施丹 shidan@tongji.edu.cn

Agree

> -----原始邮件-----
> 发件人: "施丹" <shidan@tongji.edu.cn>
> 发送时间: 2022-12-14 12:13:08 (星期三)
> 收件人: leni-dfyy <leni-dfyy@tongji.edu.cn>, "Bowen Lin" <bowenlin0512@163.com>, shenmeiting@tongji.edu.cn, lican0210@gmail.com, hugeer@tongji.edu.cn, Fu15556667713@163.com, jy279@tongji.edu.cn, "陈 磊" <mdchenlei@outlook.com>
> 抄送:
> 主题: Changes to authorship
>
> Dear co-authors,
> Manuscript Number: CDDISCOVERY-22-4968R1
> Title: PKM2 deficiency exacerbates gram-negative sepsis-induced cardiomyopathy via disrupting cardiac calcium homeostasis
>
> Considering that Can Li and Lei Chen have made adequate contributions during the revision, I would like to ask for your agreement to change/add their names in the final author lists.
> The raw order of the authors was: Le Ni, Bowen Lin, Meiting Shen, Lingjie Hu, Fengmei Fu, Can Li, Jian Yang, Dan Shi
> The final order of the authors was: Le Ni, Bowen Lin, Meiting Shen, Can Li, Lingjie Hu, Fengmei Fu, Lei Chen, Jian Yang, Dan Shi
>
> Please respond “Agree” or "Not agree" to this email.
>
> Yours sincerely,
>
> Dan Shi, MD, PhD
> Tongji University School of Medicine

发件人： leni-dfyy@tongji.edu.cn
主题： 回复：Changes to authorship
日期： 2022年12月14日 下午5:23
收件人： shidan shidan@tongji.edu.cn
抄送： bowenlin0512 bowenlin0512@163.com, shenmeiting shenmeiting@tongji.edu.cn, lican0210 lican0210@gmail.com, hugeer hugeer@tongji.edu.cn, Fu15556667713 Fu15556667713@163.com, jy279 jy279@tongji.edu.cn, mdchenlei mdchenlei@outlook.com

L

agree

leni-dfyy@tongji.edu.cn

TONGJI UNIVERSITY

邮箱：leni-dfyy@tongji.edu.cn

---- 回复的原邮件 ----

发件人 施丹<shidan@tongji.edu.cn>

日期 2022年12月14日 12:13

收件人 leni-dfyy<leni-dfyy@tongji.edu.cn>、Bowen Lin<bowenlin0512@163.com>、shenmeiting@tongji.edu.cn<shenmeiting@tongji.edu.cn>、lican0210@gmail.com<lican0210@gmail.com>、hugeer@tongji.edu.cn<hugeer@tongji.edu.cn>、Fu15556667713@163.com<Fu15556667713@163.com>、jy279@tongji.edu.cn<jy279@tongji.edu.cn>、陈 磊<mdchenlei@outlook.com>

主题 Changes to authorship

Dear co-authors,
Manuscript Number: CDDISCOVERY-22-4968R1
Title: PKM2 deficiency exacerbates gram-negative sepsis-induced cardiomyopathy via disrupting cardiac calcium homeostasis

Considering that Can Li and Lei Chen have made adequate contributions during the revision, I would like to ask for your agreement to change/add their names in the final author lists.
The raw order of the authors was: Le Ni, Bowen Lin, Meiting Shen, Lingjie Hu, Fengmei Fu, Can Li, Jian Yang, Dan Shi
The final order of the authors was: Le Ni, Bowen Lin, Meiting Shen, Can Li, Lingjie Hu, Fengmei Fu, Lei Chen, Jian Yang, Dan Shi

Please respond “Agree” or "Not agree" to this email.

Yours sincerely,

Dan Shi, MD, PhD
Tongji University School of Medicine

发件人: **lican0210** lican0210@gmail.com
主题: Re: Changes to authorship
日期: 2022年12月14日 下午6:48
收件人: 施丹 shidan@tongji.edu.cn

Agree

发自我的iPhone

------------------ Original ------------------

From: 施丹 <shidan@tongji.edu.cn>
Date: Wed,Dec 14,2022 0:13 PM
To: leni-dfyy <leni-dfyy@tongji.edu.cn>, Bowen Lin <bowenlin0512@163.com>, shenmeiting <shenmeiting@tongji.edu.cn>, lican0210 <lican0210@gmail.com>, hugeer <hugeer@tongji.edu.cn>, Fu15556667713 <Fu15556667713@163.com>, jy279 <jy279@tongji.edu.cn>, 陈 磊 <mdchenlei@outlook.com>
Subject: Re: Changes to authorship

Dear co-authors,
Manuscript Number: CDDISCOVERY-22-4968R1
Title: PKM2 deficiency exacerbates gram-negative sepsis-induced cardiomyopathy via disrupting cardiac calcium homeostasis

Considering that Can Li and Lei Chen have made adequate contributions during the revision, I would like to ask for your agreement to change/add their names in the final author lists.
The raw order of the authors was: Le Ni, Bowen Lin, Meiting Shen, Lingjie Hu, Fengmei Fu, Can Li, Jian Yang, Dan Shi
The final order of the authors was: Le Ni, Bowen Lin, Meiting Shen, Can Li, Lingjie Hu, Fengmei Fu, Lei Chen, Jian Yang, Dan Shi

Please respond “Agree” or "Not agree" to this email.

Yours sincerely,

Dan Shi, MD, PhD
Tongji University School of Medicine

发件人: **锦医18级付峰梅** fu15556667713@163.com
主题: Re: Changes to authorship
日期: 2022年12月14日 下午6:58
收件人: 施丹 shidan@tongji.edu.cn

Agree

---- Replied Message ----

From	施丹<shidan@tongji.edu.cn>
Date	12/14/2022 12:13
To	leni-dfyy <leni-dfyy@tongji.edu.cn>,<br>Bowen Lin<bowenlin0512@163.com>,<br>shenmeiting<shenmeiting@tongji.edu.cn>,<br>lican0210<lican0210@gmail.com>,<br>hugeer<hugeer@tongji.edu.cn>,<br>Fu15556667713<fu15556667713@163.com>,<br>jy279<jy279@tongji.edu.cn>,<br>陈 磊<mdchenlei@outlook.com>
Subject	Changes to authorship

Dear co-authors,
Manuscript Number: CDDISCOVERY-22-4968R1
Title: PKM2 deficiency exacerbates gram-negative sepsis-induced cardiomyopathy via disrupting cardiac calcium homeostasis

Considering that Can Li and Lei Chen have made adequate contributions during the revision, I would like to ask for your agreement to change/add their names in the final author lists.
The raw order of the authors was: Le Ni, Bowen Lin, Meiting Shen, Lingjie Hu, Fengmei Fu, Can Li, Jian Yang, Dan Shi
The final order of the authors was: Le Ni, Bowen Lin, Meiting Shen, Can Li, Lingjie Hu, Fengmei Fu, Lei Chen, Jian Yang, Dan Shi

Please respond “Agree” or "Not agree" to this email.

Yours sincerely,

Dan Shi, MD, PhD
Tongji University School of Medicine
